# Trachea, bronchus, and lung cancer in women of reproductive age: global burden trends from 1990 to 2021 and forecasts to 2035

**DOI:** 10.3389/fgwh.2026.1695260

**Published:** 2026-10-13

**Authors:** Shan Chen, Yao Rao, Yaohua Chen, Qian Yang, Guirong Wu, Fang Hu, Youshu Gao, Hongjiang Pu, Jiangping Fu

**Affiliations:** 1 Department of Respiratory and Critical Care Medicine, The Affiliated Hospital of Southwest Medical University, Luzhou, Sichuan, China; 2 Dazhou Central Hospital, Dazhou, Sichuan, China; 3 The Third Affiliated Hospital of Kunming Medical University, Peking University Cancer Hospital Yunnan Hospital, Yunnan Cancer Hospital, Kunming, China

**Keywords:** DALYS (disability-adjusted life years), incidence, mortality, the global burden (GBD), trachea, bronchus and lung cancer (TBL), women of reproductive age

## Abstract

**Background:**

In recent decades, cancers of the trachea, bronchus and lung cancer (TBL) have been the cancers with the highest global morbidity and mortality rates, with large regional variations, and the highest economic burden of all cancer types, and have shown a clear trend towards feminisation and younger onset. Therefore, we aimed to provide comprehensive insights into the prevalence and impact of TBL in women of reproductive age, taking into account factors such as age and socio-demographic indices (SDI).

**Methods:**

To assess trends in the prevalence of TBL among women of reproductive age, we used the Global Burden of Diseases, Injuries, and Risk Factors Study (GBD) 2021 to systematically assess the burden of CBD and its changes globally, regionally, and nationally from 1990 to 2021. We calculated estimated annual percentage change (EAPC), age-standardised incidence rates (ASIR), age-standardised death rates (ASDR) and disability-adjusted life years (DALYs) for 204 countries and regions from 1990 to 2021. DALYs were used as a measure in conjunction with socio-demographic indices (SDIs). Analyses include the Inequality Slope Index (ISI) and the Concentration Index (CI) to measure health inequalities, frontier analyses to estimate achievable outcomes based on levels of development, decomposition analyses to identify drivers of change in the burden of disease, analysis of yearly cyclical cohort models, and Bayesian age-period-cohort (BAPC) modelling to predict future trends in the burden of disease.

**Results:**

Compared with 1990, there was an increase in global morbidity and mortality from TBL in women of childbearing age in 2021, but there was a decrease in global morbidity and mortality, with the most pronounced downward trend in TBL in women of childbearing age in the 45–49 age group. In contrast, the global rate of DALYs increased, and the highest rate of DALYs in women of reproductive age was found in the 45–49 age group. Only low-SDI regions and medium-low-SDI regions had increased incidence and mortality rates of the 21 GBD regions, East Asia had the highest incidence and mortality rates of the 204 countries, Nigeria had the lowest incidence and mortality rates, Greenland had the highest incidence rates, and Monaco had the highest mortality rates in 2021. From 1990 to 2021, absolute and relative inequality associated with the socio-demographic index (SDI) decreases, but inequality persists. Projections of ASR for women of childbearing age from 2021 to 2035 found an overall declining trend in TBL ASIR and ASMR for women of childbearing age from 2021 onwards.

**Conclusion:**

Although the global age-standardised DALY rates for ASIR, ASDR and TBL in women of reproductive age have decreased over the past three decades, the global burden of TBL is unevenly distributed. Moreover, the prevention and control of TBL in women of reproductive age require international cooperation, multisectoral collaboration, government support, and the joint efforts of women themselves and their families and societies.

## Introduction

Trachea, bronchus and lung cancers (TBL) are the cancers with the highest global incidence and mortality rates, and TBL has the first and second highest incidence and mortality rates in men and women respectively, but with large regional variations, and the highest economic burden of all cancer types (1, 2). There is evidence of changing patterns of lung cancer, with higher incidence in young and non-smoking women, especially in Asia and countries with Asian immigrant populations (3). And some studies have already found that the incidence of lung cancer is higher in young women than in young men in the United States, and that the incidence of TBL has declined overall in both women and men aged 30–54 since 1998, but more rapidly in men (4). Tobacco is a major risk factor for TBL, with about one-quarter of TBL cases globally caused by nonsmoking factors, but the proportion of TBL caused by nonsmoking factors may be higher in some groups of women (1). Differences in the prevalence of tobacco use by gender and region affect trends in the incidence of TBL (1). Jyoti D. Patel suggests that there are significant differences in lung cancer between the sexes, that there are differences in the histologic distribution of lung cancer, that glandular differentiation is more common in females, that genetic variation may account for differences in susceptibility, that hormonal and biological factors may play a role in carcinogenesis, and that a more thorough understanding of the heterogeneity of lung cancer in different populations may lead to innovations in therapeutic and preventive strategies (5). Growing evidence suggests that lung cancer develops differently in women than in men (6). Women smokers are more likely to develop lung adenocarcinoma than men, and never-smoking women are more likely to develop lung cancer than never-smoking men; these differences are due to hormonal, genetic and metabolic differences between the sexes (5). But often women make up a minority of TBL study populations, despite evidence that they are more likely than men to benefit from TBL screening (7). Women continue to be underrepresented in lung cancer clinical trials and are often treated using data from study populations of mostly male patients, which may not be suitable for extrapolation and generalization to women, and in order to best provide targeted treatments, there is a need to increase the representation of women in lung cancer clinical trials and in further studies (8). Whereas the findings of Jemal et al. suggested an association between lung cancer and women of childbearing age: women have higher nicotine clearance than men, and premenopausal women have higher nicotine clearance than postmenopausal women, increasing the validity of women's susceptibility to smoking-related cancer risk (9). Women of childbearing age are usually defined as those aged 15–49 years (10), a life stage characterized by peak ovarian secretory function and elevated estrogen levels, which are associated with a high susceptibility to lung cancer (11). Estrogen drives lung cancer cell proliferation, angiogenesis, and remodeling of the immune microenvironment through the ERα/ERβ signaling pathway (12, 13). Epidemiological evidence suggests that premenopausal women with non-small cell lung cancer have a poorer prognosis than postmenopausal women (14); an increased number of pregnancies and children is associated with a higher risk of lung cancer, while breastfeeding has a protective effect (15); hormone replacement therapy is also associated with an increased risk of lung cancer incidence and mortality (16). The above evidence suggests that higher estrogen levels during childbearing years are an important potential factor contributing to the unique characteristics of the TBL burden in this population (17). These further emphasize that the epidemiology of women of childbearing age is critical, as early detection and treatment in this population can lead to more favorable outcomes than in older adults (18). It is therefore of practical significance to highlight the importance of the subgroup of women of childbearing age. The GBD (Global Burden of Diseases, Injuries, and Risk Factors Study) is a study on the global burden of disease, which aims to systematically assess data on diseases and injuries, and to provide a scientific basis for the formulation of health policies and the allocation of resources at the global, regional and national levels (19). Therefore, this article provides a systematic summary of the burden of TBL in women of reproductive age at the global, regional, and national levels by analyzing data from the Global Burden of Disease (GBD) 2021. Our aim was to provide comprehensive insights into the impact of TBL on morbidity, mortality, and other aspects of TBL in a specific population of women of childbearing age, taking into account factors such as age and sociodemographic indices (SDIs).

## Methods

### Data sources

GBD 2021 provides a comprehensive compilation of health-related data covering 369 diseases and 87 risk factors for 204 countries or territories for the period 1990–2021, making it the world's most extensive source of such information. In this study, we used the Global Health Data Exchange (GHDx) query tool (http://ghdx.healthdata.org/gbd-results-tool) to collect absolute and age-standardized rates of TBL-related morbidity, mortality, and DALYs among women of childbearing age globally, using the data from GBD 2021 (ASR). The information collected was further categorized by age group (15–19, 20–24, 25–29, 30–34, 35–39, 40–44 and 45–49), year, region, country or territory. In addition, the SDI, a composite indicator that combines measures of per capita income, average educational attainment and total fertility, is used as an important covariate in predicting the burden of disease and level of development in different regions. According to the SDI values, the 204 countries or territories included in the study were categorized into five groups, namely, high, medium-high, medium, medium-low and low (19).

### Statistical indicators

The study used age-standardized rates to compare health indicators across regions and countries. ASRs are calculated per 100,000 persons, adjusting for differences in age distribution to make comparisons across regions or populations more meaningful. This indicator is used in studies to analyze the incidence (ASIR), mortality (ASDR), and disability-adjusted life-years (DALYs) rates of TBL, thereby eliminating the impact of age-structural differences in the assessment of the burden of disease in order to accurately present the burden of disease in different regions and countries. To assess the temporal trends in ASR, the study utilized the EAPC method. When both EAPC and its lower 95% CI are greater than 0, the ASR is increasing; when both EAPC and its upper 95% CI are less than 0, the ASR is decreasing; otherwise, the ASR is considered to be stable over a given period of time; he larger the absolute value of EAPC, the more pronounced is the increasing or decreasing trend. With the help of this methodology, the study clearly demonstrates the trends in ASIR, ASMR and age-standardized DALYs rates of TBL in women of reproductive age globally, regionally and nationally from 1990 to 2019. This study was conducted using R software to perform all the above statistical analyses. Through the powerful data processing and analysis functions of R software, the efficient processing of complex data was realized, which ensured the accuracy and reliability of the data computation and analysis in the study, so as to draw scientific conclusions (19, 20).

### Statistical methods

#### Inequality analysis

This study employs the Slope Index of Inequality (SII) and the Concentration Index (CI) to quantify the degree of inequality in the distribution of the total burden of disease (TBL) among women of reproductive age across countries and regions with varying levels of socioeconomic development from 1990 to 2021. Both indices reflect the distribution across the entire population and are sensitive to changes in population structure, making them recommended measures for cross-national research on health inequalities (21, 22).

The SII is an absolute inequality index used to quantify the absolute difference in health outcomes between the most advantaged and least advantaged subgroups. According to the definition by Wagstaff et al., the SII is the slope of the regression line representing the relationship between the health status of a given stratum and its relative rank in the socioeconomic distribution; it can be interpreted as the absolute impact on health resulting from a move from the lowest socioeconomic group to the highest (21). The specific calculation method is as follows: Rank each country (or region) from lowest to highest based on the Socio-demographic Index (SDI), calculate each country's relative position in the cumulative population distribution (i.e., the midpoint of the cumulative population proportion), and then use this relative position as the independent variable and the age-standardized disease burden rate (e.g., DALY rate) of women of reproductive age as the dependent variable. A weighted linear regression model is then used to estimate the regression coefficient, which is defined as the SII. To address potential heteroscedasticity, robust regression methods (such as iterative reweighted least squares) are employed, assigning lower weights to observations with larger residuals in order to minimize the impact of outliers (23). The SII can be interpreted as the absolute change in the burden of disease when moving from the least developed countries (low SDI) to the most developed countries (high SDI). A negative SII indicates that a higher SDI corresponds to a lower burden of disease (i.e., the burden is more concentrated in low-SDI countries), while a positive SII indicates that the burden is more concentrated in high-SDI countries; the larger the absolute value of the SII, the greater the absolute level of inequality (23).

The CI is a measure of relative inequality calculated based on the health concentration curve; it is defined as twice the area between the concentration curve and the diagonal line. This index quantifies the extent of health inequalities associated with socioeconomic status (21). The *x*-axis of the concentration curve represents the cumulative proportion of the population sorted by SDI from lowest to highest, while the *y*-axis represents the cumulative share of the disease burden. The concentration index (CI) is defined as twice the area between the concentration curve and the 45-degree diagonal line, with values ranging from −1 to +1 (23). When the CI is positive, it indicates that the burden of disease is concentrated among people with higher socioeconomic status (referred to as “pro-rich inequality”); when the CI is negative, it indicates that the burden of disease is concentrated among people with lower socioeconomic status (referred to as “pro-poor inequality”); a CI of 0 indicates perfect equality (22). CI is not sensitive to the overall mean of health outcomes and is suitable for comparing the relative levels of inequality across different time periods or among different diseases. Similar to the SII, a negative CI indicates that the disease burden is concentrated more in low-SDI countries, while a positive CI indicates that it is concentrated more in high-SDI countries; a larger absolute value indicates a greater degree of relative inequality (23). The SII and CI are theoretically interrelated: the SII reflects absolute inequality, while the CI reflects relative inequality, and the two complement each other. Reporting both indicators provides a comprehensive picture of the absolute and relative levels of health inequality.

This study calculates both the SII and CI to comprehensively reflect cross-national inequality in both absolute and relative terms, using the age-standardized mortality rate from TBL among women of reproductive age as the health outcome measure. All analyses were conducted using R software. Estimates of the SII and CI are reported with their 95% confidence intervals, obtained using the bootstrap method or robust standard errors.

#### Frontier and decomposition analysis

To assess the relationship between the burden of TBL in women of reproductive age and the level of socio-demographic development, we constructed a frontier model based on age-standardized DALYs rates (ASDR) using SDI. Unlike traditional regression models that describe relationships between variables or predict outcomes, sophisticated statistical methods such as frontier analysis were used to explain the nonlinear relationship between SDI and disease burden and to capture the multidimensional drivers behind the burden of TBL in women of childbearing age. Frontier analysis focuses on identifying the lowest ASDR value that is theoretically achievable for each country or region based on its current level of development as a benchmark for optimal performance. This approach quantifies the gap between a country or region's current burden and its potential minimum burden, thereby identifying areas for improvement. We apply locally weighted regression (LOESS) combined with local polynomial regression using different smoothing spans (0.3, 0.4, and 0.5) to produce smooth leading lines that capture the nonlinear relationship between SDI and ASDR. To ensure the robustness of the analysis, we conducted 1,000 self-help samples and computed the mean ASDR for each SDI value. In addition, we decomposed the change in the burden of TBL among women of reproductive age from 1990 to 2021 into aging using the Das Gupta decomposition methodology, contributions of population growth and epidemiologic changes. This approach allows us to decompose the overall change in burden into these key factors, providing a clearer understanding of how demographic and epidemiologic changes shape trends over time. Unlike traditional methods such as linear regression, which focus primarily on establishing relationships between variables, decomposition analysis enables a detailed assessment of the independent contribution of each factor to overall changes in the burden of disease. By dissecting these trends, we gain a clearer understanding of the underlying drivers of changes in the global burden of congenital birth defects.

#### BAPC model predictions

This study employs the Bayesian Age-Period-Cohort (BAPC) model to forecast future disease burden. Built upon the Bayesian generalized linear model framework, this model assumes that the number of cases follows a Poisson distribution. It decomposes the logarithm of the hazard rate into population intercept, age effect, period effect, and cohort effect, and allows for the inclusion of excess-discrete random effects; In this model, the age, period, and cohort effects are assumed to evolve dynamically and are smoothed using a second-order random walk (RW2), thereby enabling the capture of nonlinear time trends and linear extrapolation (24–26).

Model inference employs Integrated Nested Laplace Approximation (INLA), a deterministic Bayesian computational method capable of quickly and accurately approximating posterior marginal distributions. This approach effectively avoids the mixing and convergence issues commonly associated with Markov chain Monte Carlo methods while maintaining computational efficiency (24, 27). The specific computational workflow of INLA is as follows: First, approximate the marginal posterior distributions of the hyperparameters—since hyperparameters are typically related to the precision of random effects (such as age, period, and cohort effects)—INLA first identifies the mode of these hyperparameter posterior distributions, then uses methods such as centered composite designs to select a set of nodes centered on this mode for numerical integration, and finally obtains the marginal posterior distributions of the hyperparameters via interpolation functions; Second, the posterior marginal distributions of the model parameters are computed—INLA offers three strategies: Gaussian approximation, full Laplace approximation, and simplified Laplace approximation. Among these, the simplified Laplace approximation is the default method in the R-INLA package due to its balance of efficiency and accuracy; finally, the distributions of the hyperparameters and the model parameters are combined via numerical integration to obtain the posterior marginal distributions of all parameters of interest (27). Furthermore, INLA fully leverages the conditional independence among variables in latent Gaussian models and the computational advantages of sparse matrices, making it far more computationally efficient than traditional MCMC methods. By setting future observations as missing, the model automatically extrapolates the time series and cohort effects based on an RW2 prior. The prediction variance increases with the prediction step size, and the mean and variance of the prediction distribution are calculated directly, ultimately outputting age-specific and age-standardized incidence rates and case counts. When analyzing geographic distribution, spatial structure effects (conditional autoregressive priors) and spatiotemporal interaction effects can be further incorporated into this framework to capture unobserved spatiotemporal heterogeneity (28).

This study utilized data from the Global Burden of Disease (GBD 2021) study and population projections from the Institute for Health Metrics and Evaluation (IHME) to estimate the global burden of TBL among women of reproductive age using the BAPC R package, taking into full account the complex interactions among age, period, and cohort effects.

## Results

### Global burden trends

#### Incidence

According to the large-scale data analysis of the GBD database, the global prevalence trend of TBL in women of childbearing age shows significant dynamic changes, with the incidence rate increasing and then decreasing, with the peak incidence rate in 2001, with the fastest rate of decline in the 2010–2016 phase, and the incidence rate of TBL in women of childbearing age in 2021 is already significantly lower than that in 1990 (Figure 1A). The global incidence of TBL in women of reproductive age was 30,716 cases (95% UI, 27700.813–34376.797) in 1990 and 45,858.743 cases (95% UI, 40305.545–52605.496) by 2021. Although the number of cases of TBL in women of childbearing age has increased, the incidence rate did decrease, from 2.726 cases per 100,000 (95% UI, 2.464–3.046) in women of childbearing age in 1990 to 2.234 cases per 100,000 (95% UI, 1.964–2.563) in women of childbearing age in 2021. This EAPC was −0.811% (95% UI, −1.001–0.620) (Supplementary Table S1). It is worth noting that the number of TBL incidence cases in women of childbearing age aged 35–49 years has increased in 2021 compared to 1990, with the largest increase in the number of TBL incidence cases in women of childbearing age in the 45–49 year age group. However, in contrast to the incidence rate, the incidence of TBL in women of reproductive age in the 35–49 year age group in 2021 has instead declined compared to 1990, with the most pronounced decline in women of reproductive age in the 45–49 year age group. The number of TBL cases increases as the age of women of childbearing age increases, with the highest number of TBL cases occurring in women of childbearing age between 45 and 49 years old (Figure 2A).

**Figure 1:**
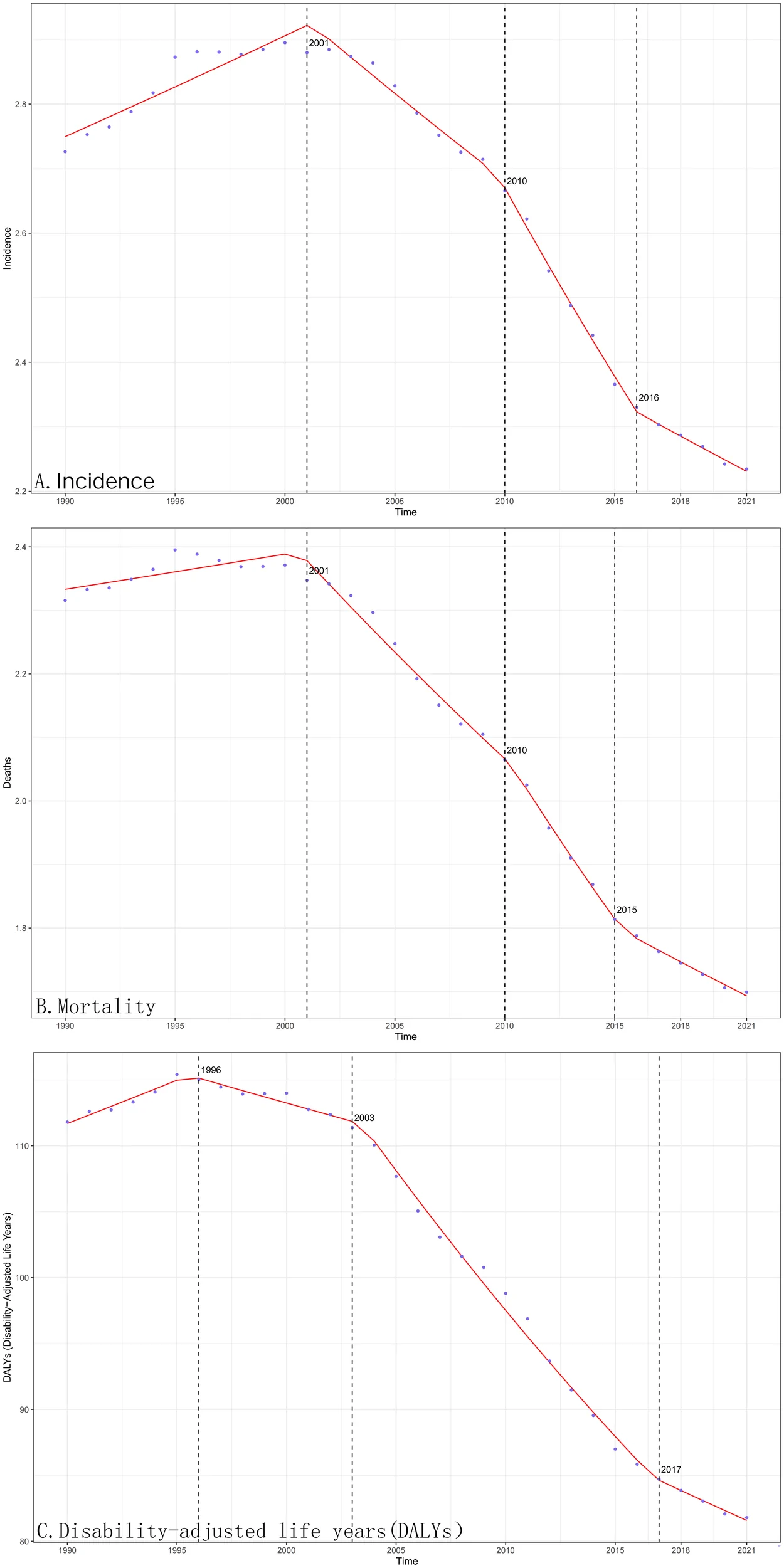
AAPC for TBL in women of reproductive age.

**Figure 2:**
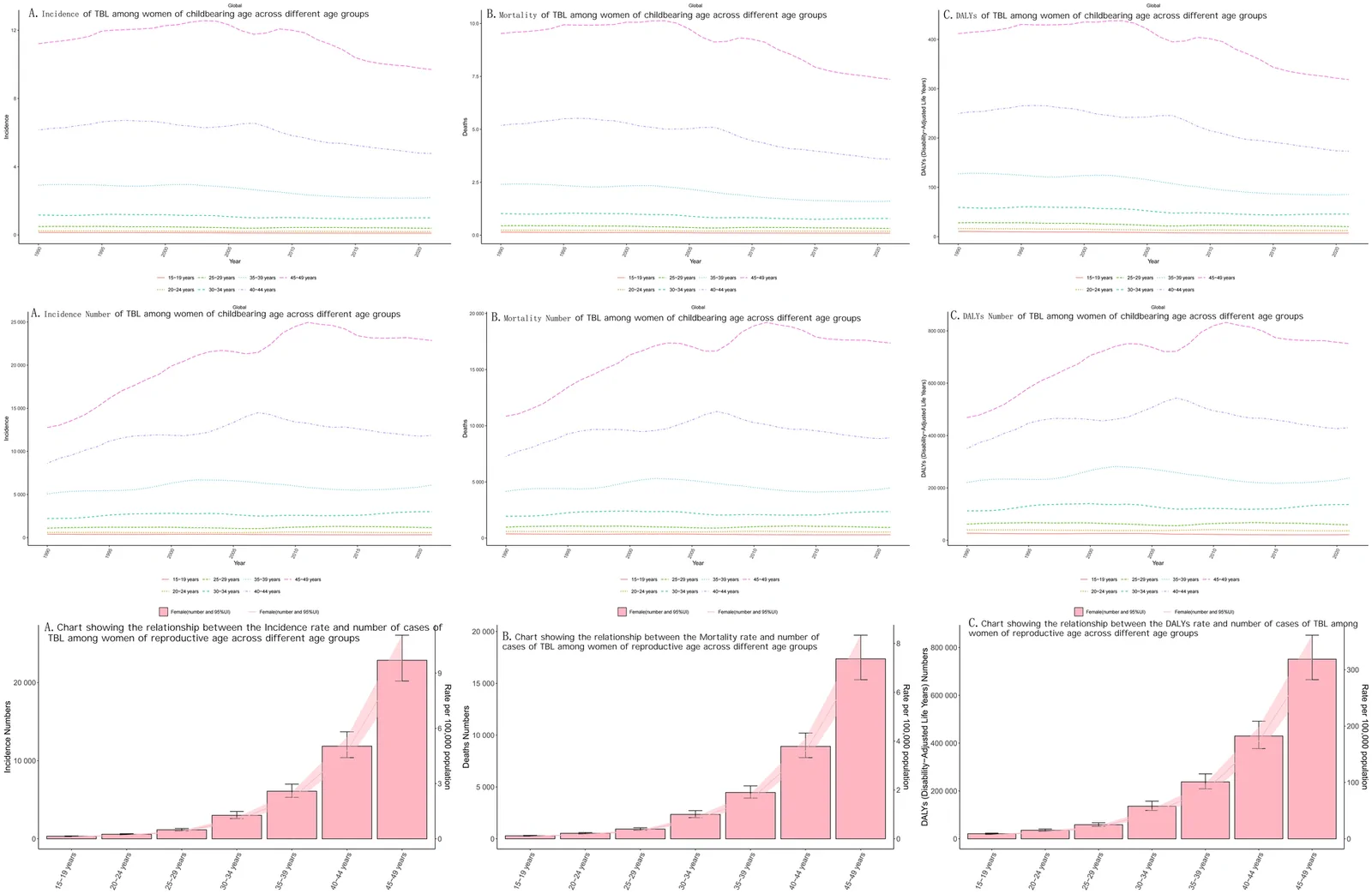
Incidence, mortality, and DALYs of TBL among women of reproductive age across different age groups. **(A)** Incidence of TBL among women of reproductive age across different age groups. **(B)** Mortality of TBL among women of reproductive age across different age groups. **(C)** DALYs of TBL among women of reproductive age across different age groups.

#### Mortality

Mortality rates are similar to morbidity rates, with mortality rates associated with TBL in women of childbearing age generally trending upward and then downward over the past 30 years, with mortality rates peaking in 2000, with the fastest rate of decline in the 2010–2016 period, and mortality rates for TBL in women of childbearing age already well below 1990 mortality rates by 2021 (Figure 1B). Globally, the number of TBL deaths in women of reproductive age was 26,126.0 (95% UI, 23,408.183–29,363.587) in 1990 and 34,856 (95% UI, 30,701.368–3,960.427) by 2021, while the mortality rate decreased from 2.316 per 100,000 in 1990 (95% UI, 2.079–2.598) to 1.699 cases per 100,000 in 2021 (95% UI, 1.496–1.935). This EAPC is −1.212% (95% UI, −1.387–−1.036) (Supplementary Table S2). Similar to the incidence rate for TBL deaths among women of reproductive age, the number of deaths in 2021 compared to 1990 also increased for women of reproductive age in the 35–49 age group and remained almost unchanged for the rest of the age group. The largest increase in the number of TBL deaths among women of reproductive age is in the 45–49 age group. Similarly, the TBL mortality rate for women of childbearing age 35–49 years has declined, with the most pronounced decline in women of childbearing age 45–49 years. The number of TBL deaths in women of reproductive age also increased with age, with the highest number of TBL deaths occurring in women of reproductive age 45–49 years (Figure 2B).

#### Disability-adjusted life years

Consistent with morbidity and mortality trends, TBL-associated disability in women of childbearing age has generally trended upward and downward over the past 30 years, with DALYs peaking in 1996, with the largest APCs and the fastest declines in their DALYs from 2003 to 2017. The DALYs in 2021 are much smaller than those in 1990 (Figure 2C). In 1990, the global DALYs for TBL in women of reproductive age were 1280027.078 (95% UI, 1144726.553–1441354.655), while in 2021, the global DALYs increased to 1671409.087 (95% UI, 1472330.392–1904819.043). The rate of DALYs decreased from 111.803 (95% UI, 100.210–125.630) per 100,000 in 1990 to 81.792 (95% UI, 72.040–93.224) per 100,000 in 2021. This EAPC is −1.229% (95% CI, −1.397–1.060) (Supplementary Table S3). For different age groups of women of childbearing age, DALYs increased in 2021 compared to 1990 for ages 40–49 and increased with age, with the highest DALYs for women of childbearing age in the 45–49 age group. Whereas the rate of DALYs declined in the 35–49 age group, there was little change in the remaining age groups (Figure 2C).

### Trends in TBL among women of childbearing age in the SDI region

Compared with 1990, the number of morbidity and mortality among women of childbearing age in 2021 increased in all four SDI regions except for the high SDI region, where the number of women of childbearing age in 2021 decreased (Figures 3A, B; Supplementary Tables S1, S2), and in terms of morbidity and mortality, the number of women of childbearing age in 2021 increased only in the low SDI region and the medium-low SDI region, while the number of women of childbearing age in 2021 declined in the other three SDI regions, and declined most rapidly in the high SDI region from about 2010 onward. The highest rate of decline was observed in the high SDI region from around 2010 onwards (Figures 3A, B; Supplementary Tables S1, S2). In addition, the EAPC associated with the incidence of TBL in women of childbearing age was highest in the low and middle SDI regions at 0.747% (95% CI, 0.692–0.802) and lowest in the high SDI regions at −1.345% (95% CI, −1.783–0.904) (Supplementary Table S1). As for the EAPC associated with TBL mortality in women of childbearing age, again the EAPC of 0.680% (95% CI, 0.625–0.736) was the highest in the medium-low SDI region, and the EAPC of −2.007% (95% CI, −2.389–1.622) was the lowest in the high SDI region (Supplementary Table S2). DALYs were similar to morbidity and mortality, with TBL DALYs declining only in high SDI areas and DALYs rates increasing only in low SDI areas and medium-low SDI areas in women of childbearing age in 2021 compared with 1990 (Figure 3C; Supplementary Table S3). Among the EAPCs associated with rates of TBLDALYs in women of childbearing age, the highest was 0.662% (95% CI, 0.605–0.720) in the medium-low SDI region, and the lowest was −1.995 (95% CI, −2.361–1.627) in the high SDI region (Supplementary Table S2).

**Figure 3:**
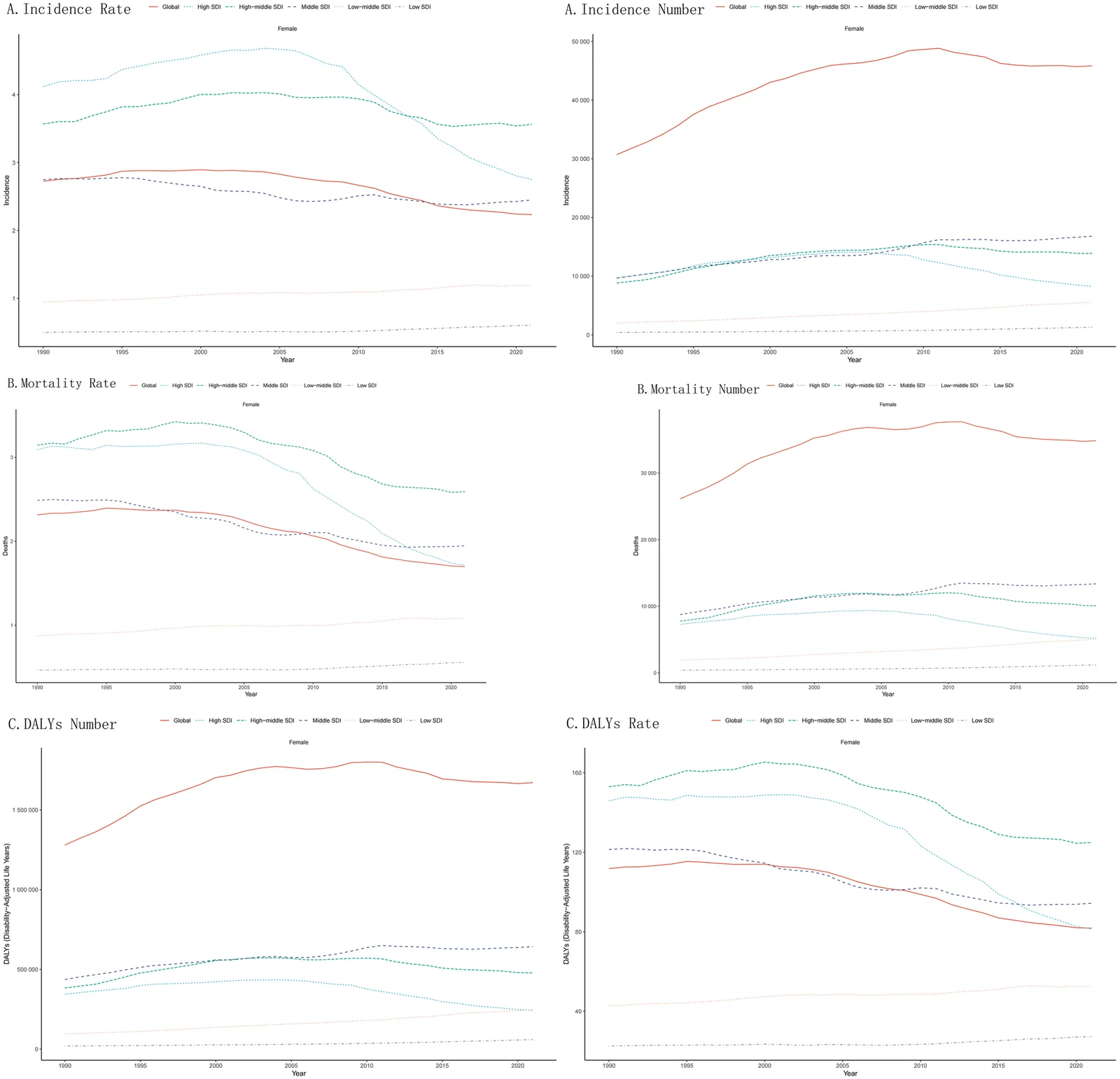
Trends in the incidence, mortality, and DALYs of TBL among women of childbearing age in different SDI regions, 1990–2021.

### Burden of disease in 21 GBD regions

#### Incidence

Analysis of the burden of disease for TBL incidence among women of childbearing age in 21 GBD districts in 2021 found that the highest incidence in all 21 districts was among women of childbearing age in the 45–49 year age group, and that the vast majority of them accounted for more than 50% of the cases (Figure 4). In 1990, among the 21 GBD regions, the highest incidence of TBL in women of childbearing age was 6.245 cases per 100,000 in High-income North America (95% UI, 5.982–6.494), and the lowest was 0.243 cases per 100,000 in Western Sub-Saharan Africa (95% UI, 0.186–0.316). By 2021, the highest incidence rate became 4.749 cases per 100,000 people in East Asia (95% UI, 3.581–6.214), and the lowest remained 0.280 cases per 100,000 people in Western Sub-Saharan Africa (95% UI, 0.194–0.380) (Supplementary Table S1). As for the EAPC associated with the incidence of TBL in women of childbearing age, South Asia EAPC1.528% (95% CI, 1.381–1.676) was the highest, and High-income North America EAPC −2.832% (95% CI, −3.363–2.298) was the lowest (Supplementary Table S1).

**Figure 4:**
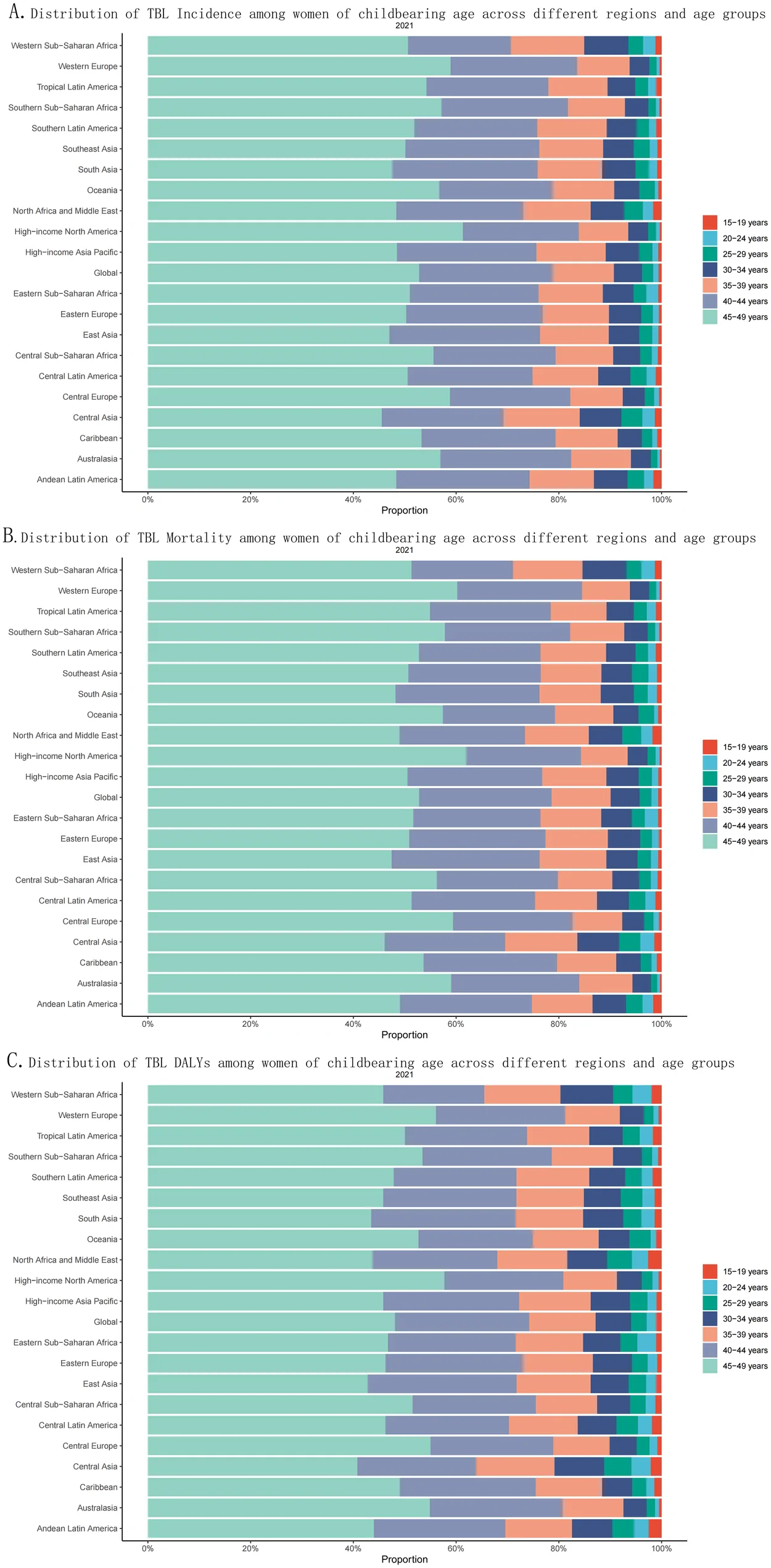
Distribution of TBL incidence, mortality and DALYs among women of childbearing age across different regions and age groups in 2021.

#### Mortality

The highest burden of disease for TBL mortality among women of childbearing age in the 21 GBD regions of all ages was among women of childbearing age in the 45–49 age group, and most of them accounted for more than 50% of the burden of disease (Figure 4). At 1990 and 2021, the 21 GBD regions with the highest TBL mortality rates among women of reproductive age were both East Asia, with rates of 4.206 cases per 100,000 people (95% UI, 3.311–5.261) and 3.388 cases per 100,000 people (95% UI, 2.549–4.426) respectively; the lowest rates were both Western Sub-Saharan Africa, with mortality rates of 0.22 per 100,0005 (95%UI, 0.172–0.294) and 0.257 per 100,000 (95%UI, 0.179–0.350) respectively (Figure 4). Its EAPC associated with TBL mortality in women of reproductive age was highest in South Asia EAPC 1.440% (95% CI, 1.299–1.582) and lowest in High-income North America EAPC −3.221% (95% CI, −3.695–2.746) (Supplementary Table S1). In addition, the overall trend in TBL mortality among women of childbearing age in the 21 GBD districts was an increase and then a decrease with increasing SDI (Figure 5).

**Figure 5:**
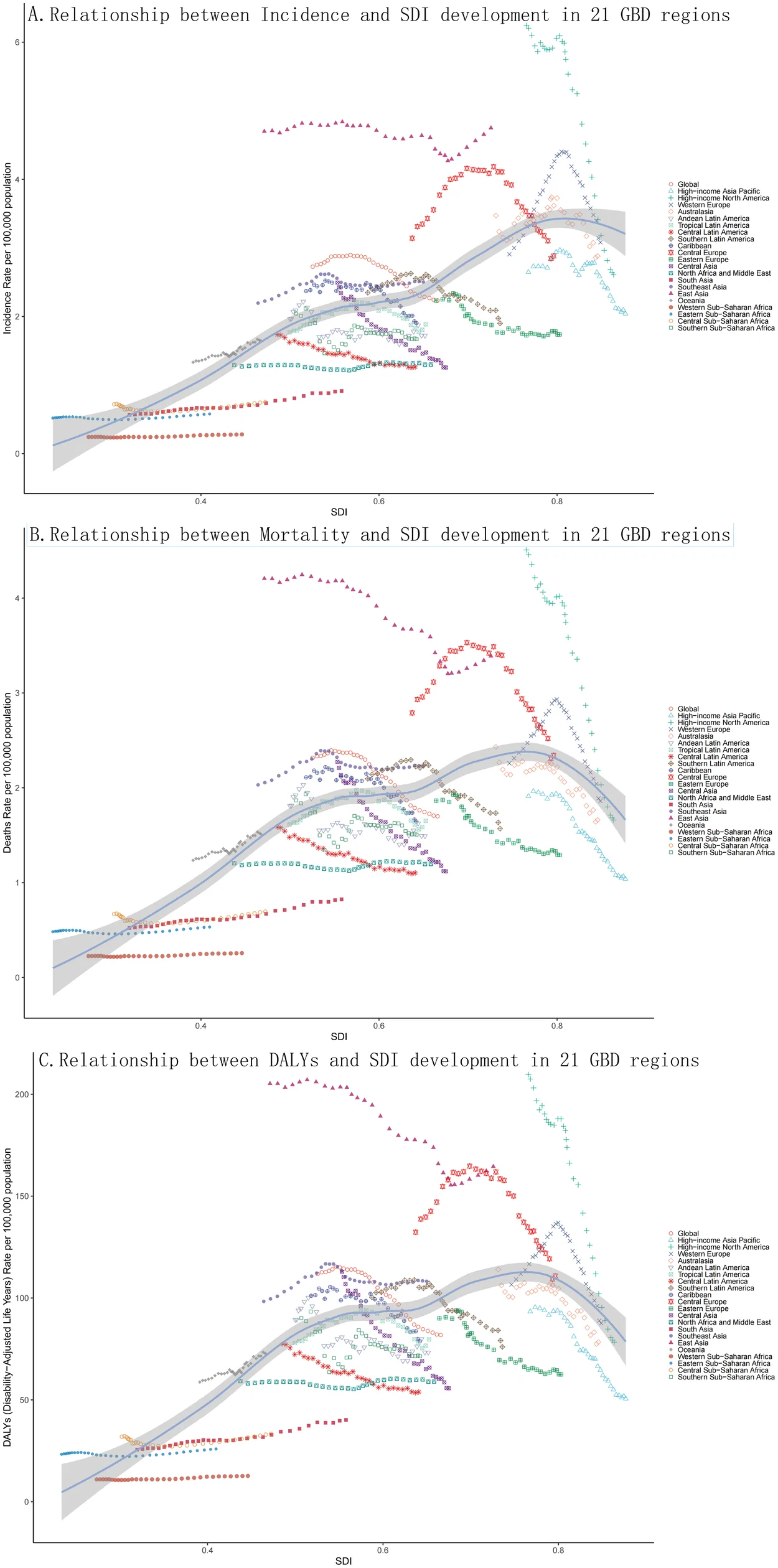
Relationship between incidence, mortality, and DALYs and SDI development in 21 GBD regions.

#### Disability-adjusted life years

As with morbidity and mortality, the highest proportions of the burden of disease for TBL mortality among women of childbearing age in all 21 GBD regions continued to be among women of childbearing age in the 45–49 age group (Figure 4). The highest rate of TBLDALYs among women of childbearing age was 209.704 (95% UI, 201.403–216.561) per 100,000 in High-income North America in 1990, and the highest in 2021 was 164.539 (95% UI, 123.766- 215.196). In contrast, the lowest rates of TBLDALYs among women of childbearing age in both 1990 and 2021 were in Western Sub-Saharan Africa, at 10.969 per 100,000 (95% UI, 8.345–14.341) and 12.698 per 100,000 (95% UI, 8.781–17.344) respectively (Supplementary Table S3). And the highest and lowest EAPC associated with rates of TBLDALYs in women of childbearing age remained South Asia EAPC 1.416% (95% CI, 1.284–1.547) and High-income North America EAPC −3.209 (95% CI, −3.668–2.748) (Supplementary Table S1). Similar to morbidity and mortality, the overall trend in the rates of TBL DALYs among women of reproductive age in the 21 GBD districts continued to increase and then decrease with increasing SDI (Figure 5).

### Burden of disease in 204 countries

#### Incidence

According to the GBD database, among the 204 countries, Nigeria had the lowest incidence of TBL in women of reproductive age in both 1990 and 2021, with rates of 0.082 (95% UI, 0.047–0.130) and 0.116 (95% UI, 0.068–0.189) cases per 100,000 persons, respectively; whereas the highest incidence rate in Denmark in 1990 was 6.891 cases per 100,000 people (95% UI, 5.728–8.155) to 7.936 cases per 100,000 people in Greenland in 2021 (95% UI, 4.965–12.265) (Supplementary Tables S4; Figure 6). As for the number of incidence of TBL in women of reproductive age, China leads the way due to its large population, both in 1990 and 2021 (Supplementary Tables S4; Figure 6). EAPCs associated with the incidence of TBL in women of childbearing age in 204 countries were fastest growing for Lesotho EAPC 4.218% (95% CI, 3.508–4.933) and fastest declining for Kuwait EAPC −3.388% (95% CI, −4.169–2.600) (Supplementary Tables S4; Figure 6). In addition, analyzing the relationship between the SDI and the incidence of TBL in women of childbearing age in 204 countries, it was found that countries with a higher SDI had a higher incidence of TBL in women of childbearing age (Figure 9A).

**Figure 6:**
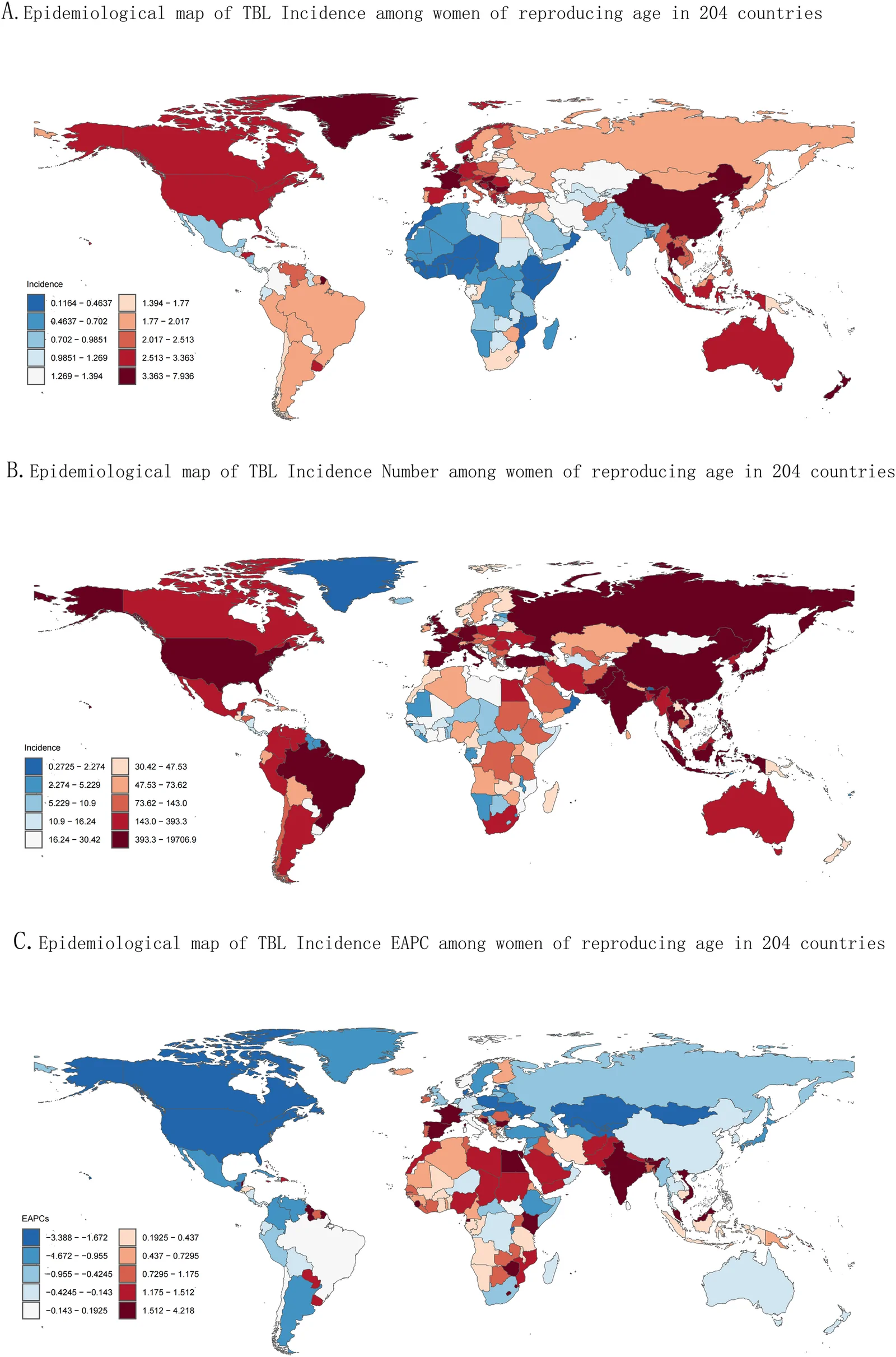
Epidemiological map of TBL incidence among women of reproducing age in 204 countries.

#### Mortality

Analysis of 1990 and 2021 data from 204 countries found that Nigeria likewise had the lowest TBL mortality rates among women of reproductive age, with rates of 0.076 cases per 100,000 (95% UI, 0.043–0.121) and 0.106 cases per 100,000 (95% UI, 0.063–0.171), respectively; and by 1990 Denmark topped the list with a mortality rate as high as its incidence rate, at 5.240 cases per 100,000 (95% UI, 4.395–6.127), while the country with the highest mortality rate in 2021 was Monaco at 7.647 per 100,000 (95% UI, 4.947–11.349) (Supplementary Table S5; Figure 7). And China ranks first in the number of deaths both in 1990 and 2021, out of 204 countries (Supplementary Table S5; Figure 7). For EAPCs associated with TBL mortality in women of childbearing age, the fastest growing was the Lesotho EAPC 3.409% (95% CI, 2.346–4.483), and the fastest declining was the Canada EAPC −4.020% (95% CI, −4.494–−3.543) (Supplementary Tables S5; Figure 7). And the association between national SDIs and female mortality in the reproductive age group is similarly such that countries with higher SDIs have higher TBL mortality rates among women in the reproductive age group (Figure 9B).

**Figure 7:**
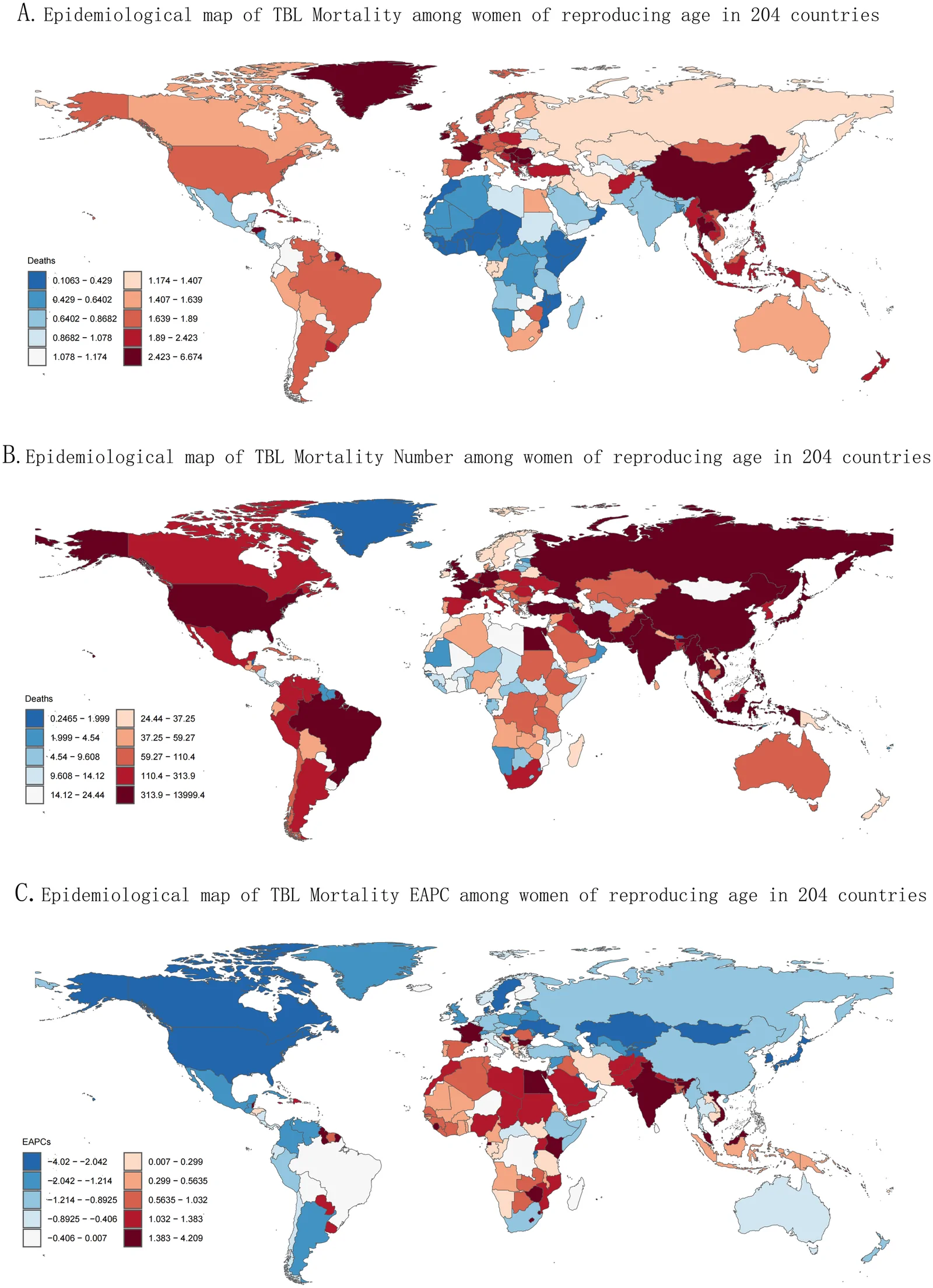
Epidemiological map of TBL mortality among women of reproducing age in 204 countries.

#### Disability-adjusted life years

Using the data, it can be seen that the state with the lowest rate of DALYs in both 1990 and 2021 was Nigeria, with DALYs rates of 3.636 (95% UI, 2.072–5.837) per 100,000 people and 5.129 (95% UI, 3.021–8.327) per 100,000 people respectively; whereas the state with the highest rate of DALYs was both Greenland, with DALYs rates of 543.059 (95% UI, 368.451–767.705) per 100,000 and 309.511 (95% UI, 186.796–481.104) per 100,000, accordingly (Supplementary Table S6; Figure 8) China's DALYs still top the list of 204 countries (Supplementary Table S6; Figure 8). EAPCs associated with rates of TBLDALYs in women of childbearing age, the fastest growing was Lesotho EAPC 4.232% (95% CI, 3.518–4.952) the fastest declining was Canada EAPC −3.985% (95% CI, −4.433–3.535) (Supplementary Table S6; Figure 8). Similarly countries with higher SDIs have higher rates of TBLDALYs in women of childbearing age (Figure 9C).

**Figure 8:**
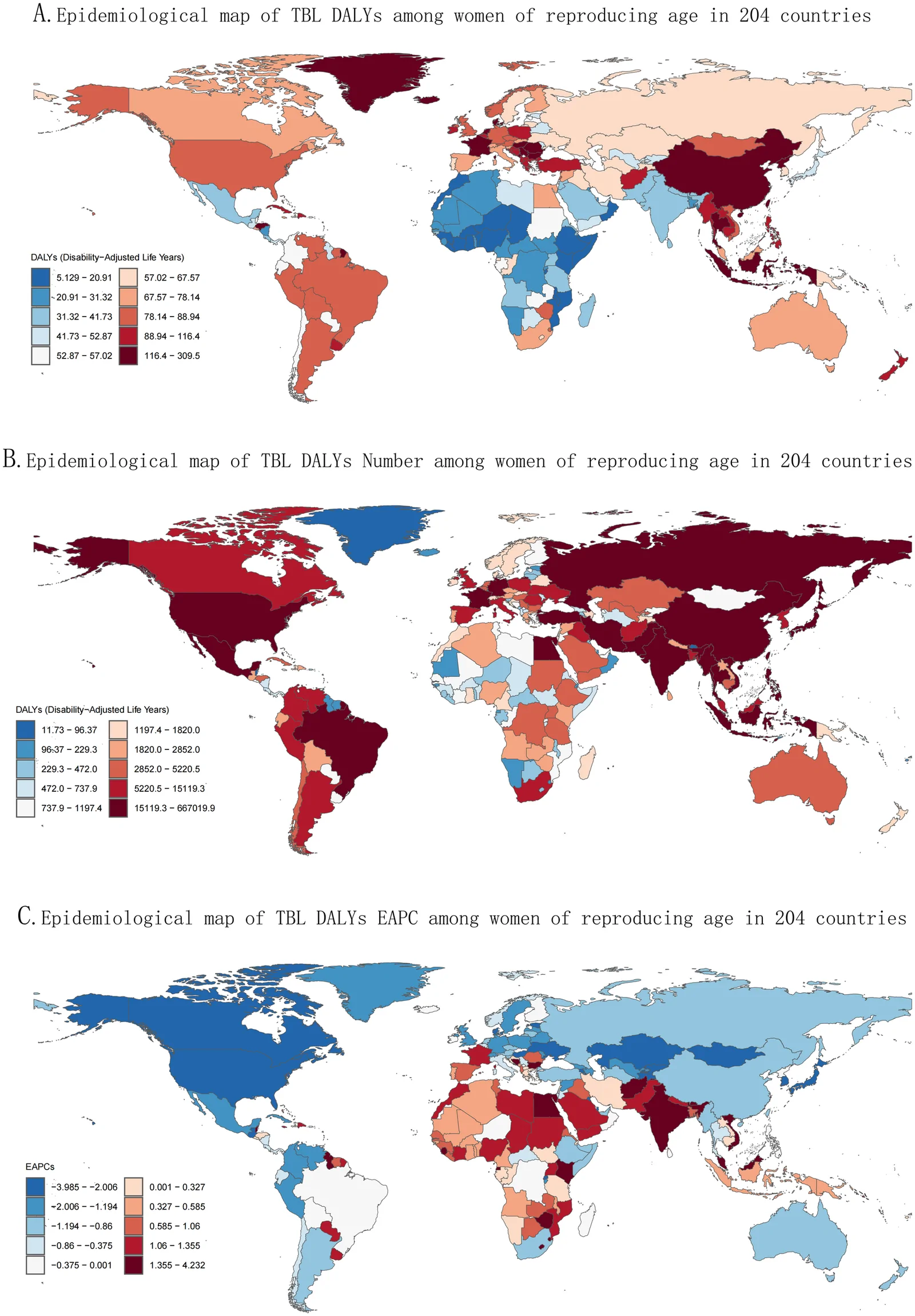
Epidemiological map of TBL DALYs among women of reproducing age in 204 countries.

**Figure 9:**
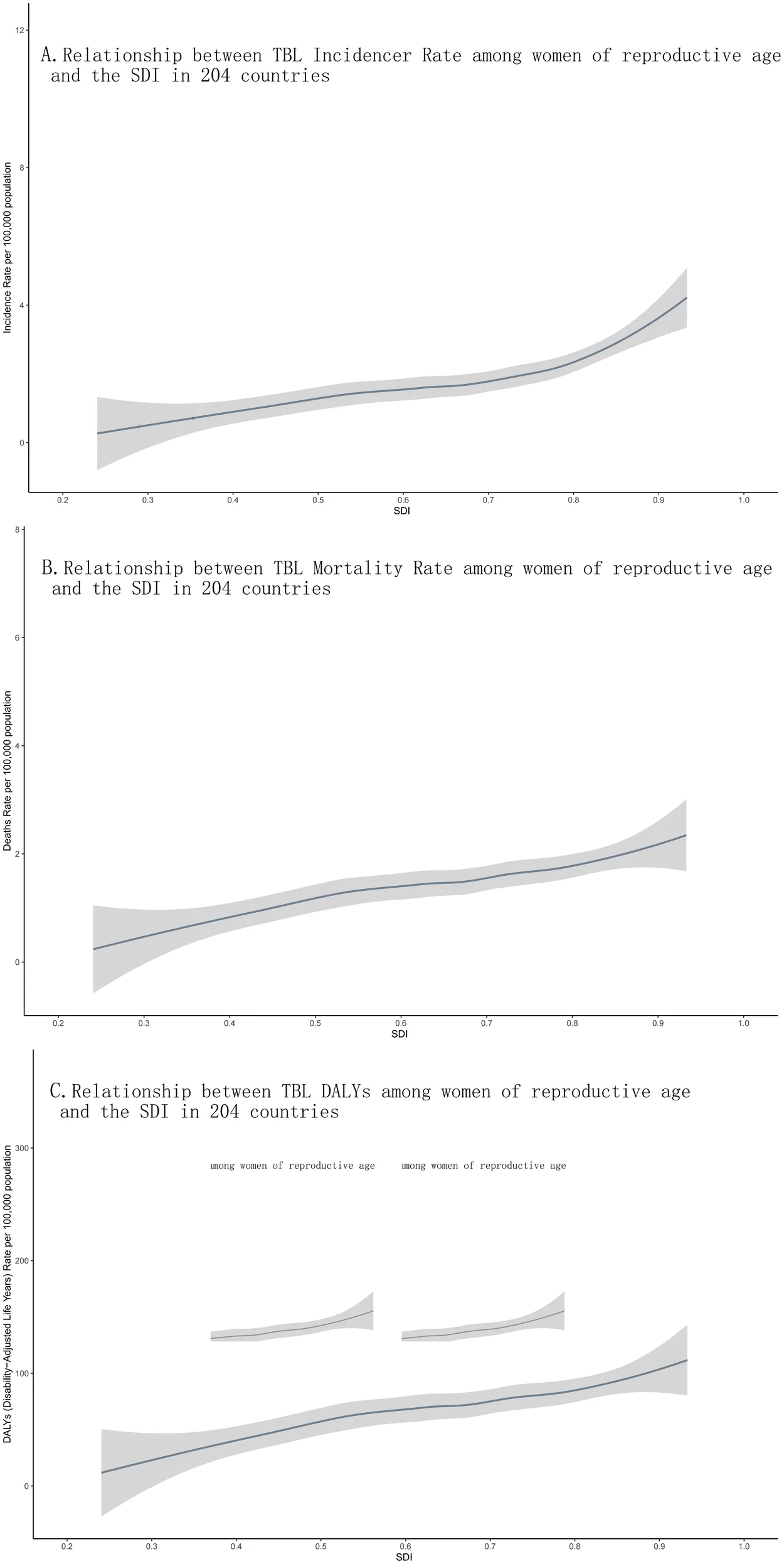
Relationship between TBL incidence, mortality, and DALYs among women of reproductive age and the SDI in 204 countries.

### Inequality analysis

Regarding the burden of TBL for women of childbearing age, we observe a reduction in absolute and relative inequality associated with the sociodemographic index (SDI), but inequality persists (Figure 10, *p* < 0.05). The concentration index CI is 0.14 (95% UI, −0.09–0.53) in 1990, but declines to 0.11 (95% UI, −0.07–0.25) in 2021 (Figure 10). Overall, both the cumulative proportion of deaths in 2021 and its range of uncertainty have narrowed, which may suggest that the influence of relevant factors on mortality scenarios has moved in a more stable and potentially more favorable direction over the 1990–2021 period, suggesting that global inequality in TBL for women of childbearing age remains a persistent problem, although the gap between the rich and the poor has narrowed in some regions.

**Figure 10:**
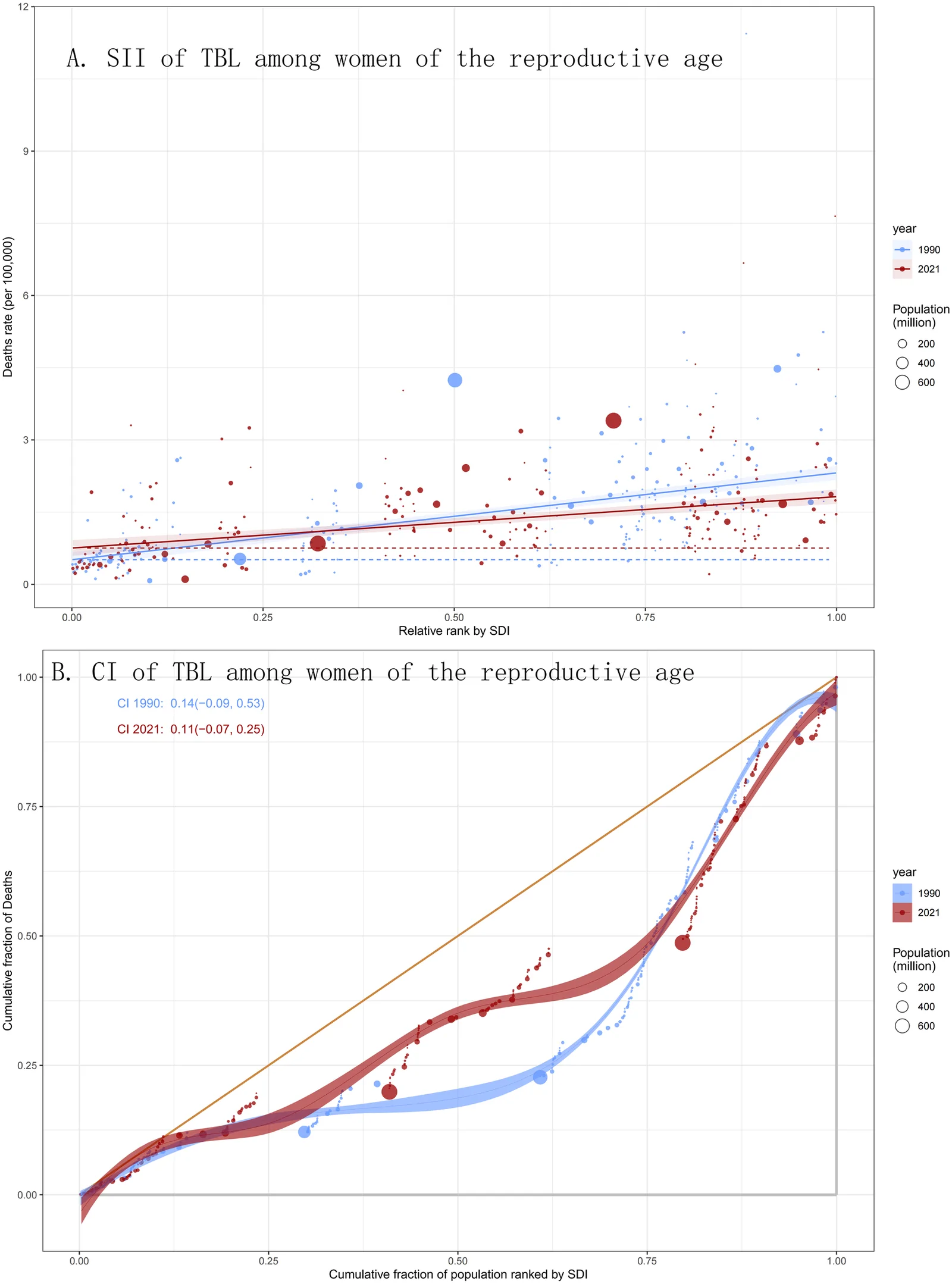
Analysis of inequalities in the disease burden of TBL among women of reproductive age.

### Age-cycle cohort modeling analysis

We analyzed the association between women of reproductive age and the prevalence of TBL in different age cohorts using data from the GBD database. The prevalence of TBL in women of reproductive age increased with age (Figure 11IB). Trends in the prevalence of TBL among women of childbearing age by period showed a generally decreasing trend with increasing years (Figure 11IC). An emergence cohort was calculated to assess the prevalence of TBL in women of childbearing age and found a broadly decreasing trend with increasing birth year (Figures 11IA, D). From the images, it can be seen that the prevalence of women of childbearing age in each birth cohort showed a significant increase with age (Figure 11IIA); and different birth cohorts showed different levels of prevalence at the same age stage, with earlier birth cohorts of women of childbearing age (e.g., the 1940 birth cohort) showing a relatively high prevalence of TBL as they moved into the higher age groups (e.g., 40 years and over) higher; whereas later birth cohorts (e.g., 2000 birth cohort) have a relatively lower prevalence at younger ages (15–29 years) (Figure 11IIB).

**Figure 11:**
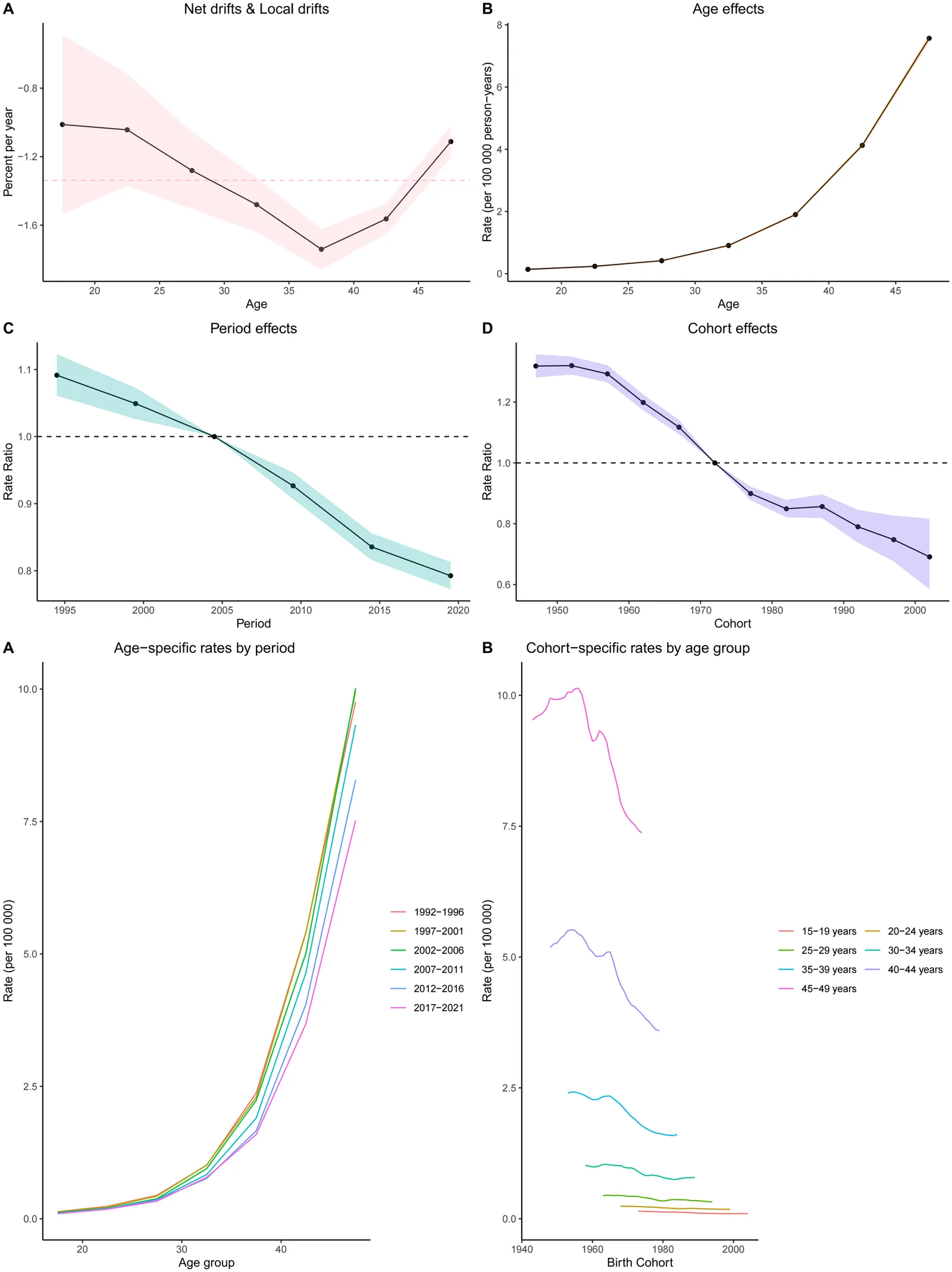
Age-group analysis of TBL in women of reproducing age.

### Decomposition analysis

The global prevalence of TBL in women of reproductive age has increased significantly over the past 30 years, mainly due to population growth and population aging, while epidemiological changes have had a negative impact on prevalence. Among the quintile regions, the middle SDI region had the largest increase in the incidence of TBL in women of childbearing age. East Asia had the largest increase in the incidence of TBL in women of reproductive age, mainly due to population growth (Figure 12). Whereas the effects of population aging, demographics, and epidemiology on the rates of TBL mortality and DALYs in women of reproductive age are similar to those of morbidity, for global and regional (Figure 12).

**Figure 12:**
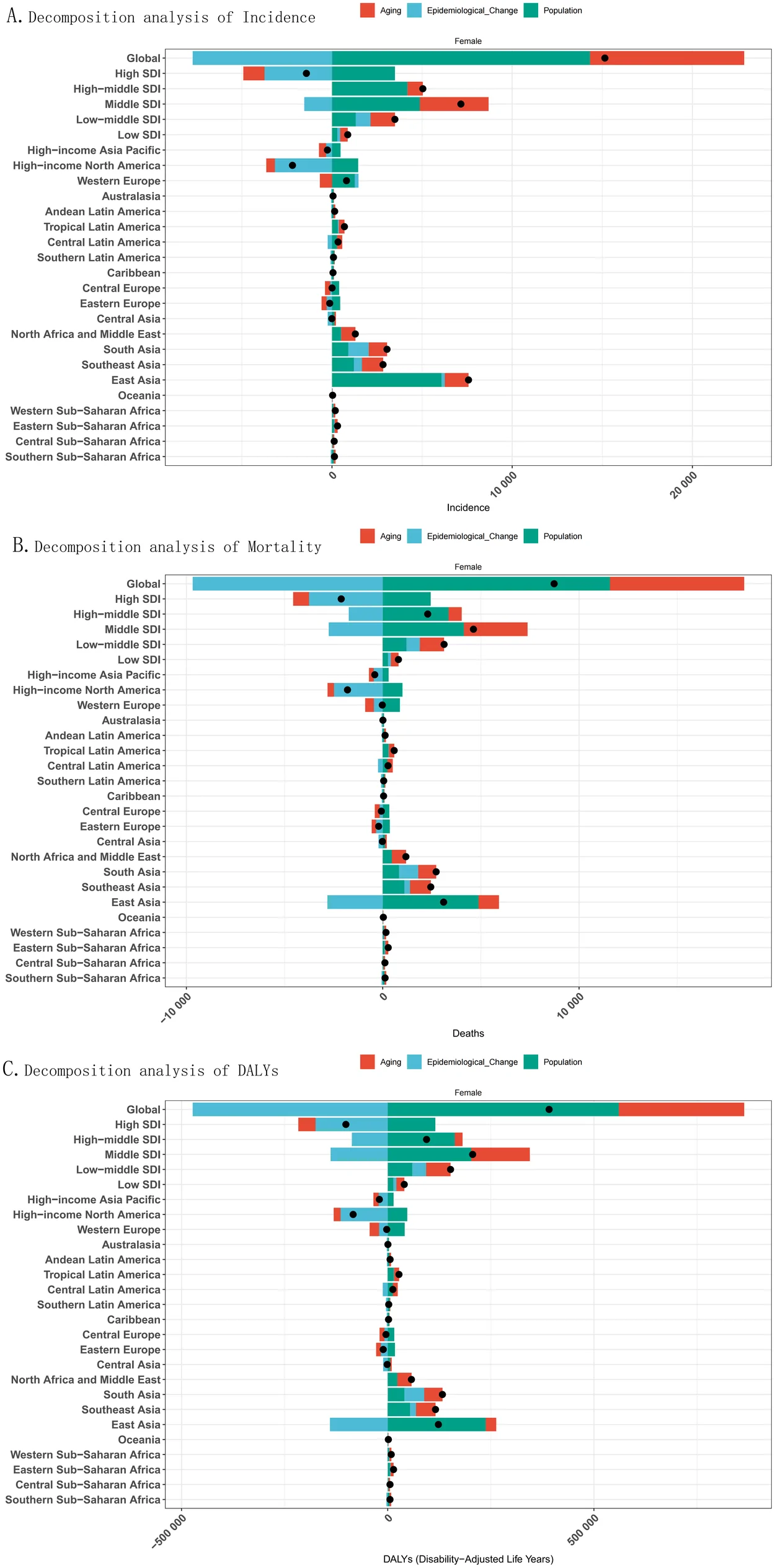
Decomposition analysis of the disease burden of TBL among women of reproducing age.

### Forward analysis

Using data from 1990 to 2021, based on ASIR, ASMR, ASDR, and SDI, a frontier analysis was conducted to explore the potential room for improvement in ASIR, ASMR, and ASDR of TBL in women of childbearing age, taking into account the level of development of the country and region. From 1990 to 2020, some countries or regions, such as Monaco and Iceland, show a decreasing trend in mortality rates over time, indicating that these places have been effective in improving the health of their populations and reducing the risk of death, while some countries or regions, such as Somalia and the Niger, show an increasing trend, which may be related to local sanitary conditions, the prevalence of diseases, social unrest and other factors. On the whole, there is a trend of gradual reduction in mortality rates as the SDI increases, indicating that socio-economic development has had a positive effect on the reduction of population mortality rates, but there are a few exceptions, such as the fact that mortality rates are still higher in certain areas with higher SDIs, probably because these areas face some special health challenges, such as the increase in mortality rates for chronic diseases due to serious ageing (Figure 13B). Countries or regions like Monaco and Iceland have seen a downward trend in the rate of DALYs, which may mean that these places have made progress in disease prevention, treatment, and rehabilitation reducing the overall impact of diseases and injuries on the health of the population; whereas countries or regions like Somalia and Niger have seen an increase in the rate of DALYs, which may be due to the prevalence of infectious diseases, insufficient healthcare resources, and social instability that have led to a disease burden that is Aggravation. Overall, there is a trend of decreasing DALYs rates as the SDI rises, reflecting the fact that socio-economic development has helped to reduce the health burden of diseases and injuries, but there are also some areas with higher SDIs where DALYs rates are still high, which may be due to the fact that there are some intractable health problems in these areas such as the high prevalence of chronic diseases and the prominence of mental health problems, which need to be further strengthened by targeted public health interventions (Figure 13C). Countries or regions such as Iceland and the Netherlands show a downward trend in incidence rates, which may be related to factors such as the strengthening of disease prevention measures, advances in medical technology and the popularization of health education in those places; while countries or regions such as Somalia and the Niger show an increase in incidence rates, which may be attributed to poor sanitary conditions, insufficient capacity for disease surveillance and prevention and control, and the increased risk of disease transmission as a result of population growth and mobility. Overall, the relationship between morbidity and SDI is more complex, with no clear pattern of decreasing with increasing SDI as in the case of mortality and DALYs rates. Some areas with higher SDIs have lower morbidity rates, probably because they have a well-developed public health system that can effectively control the occurrence of diseases; however, there are also areas with higher SDIs that have higher morbidity rates, which may be due to the fact that they are facing some new health threats, such as the high prevalence of lifestyle diseases (e.g., obesity diabetes mellitus, etc.), or the increase in the incidence of chronic diseases due to the aging of the population, etc., which need to be analyzed according to the reasons behind this need to be analyzed according to the specific conditions of different regions and appropriate health strategies need to be adopted to cope with it (Figure 13A).

**Figure 13:**
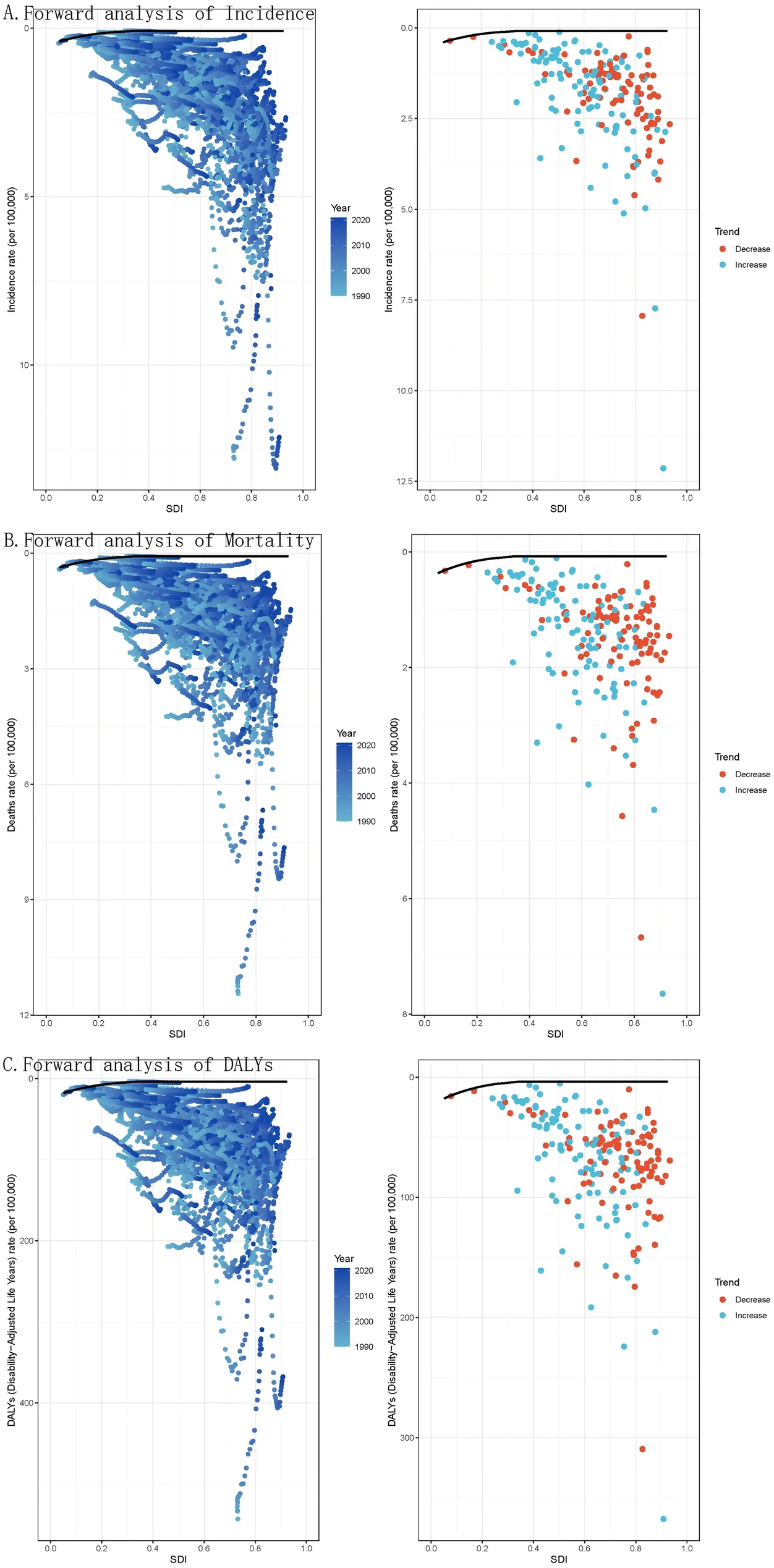
Forward analysis of the disease burden of TBL among women of reproducing age.

### BAPC

To understand the global ASR trend of TBL for women of reproductive age after 2021, we used Bayesian age-period-cohort modeling to project the ASR for women of reproductive age from 2021 to 2035, and found that the ASIR of TBL for women of reproductive age has been on an overall downward trend from 2021 to 2035, but an overall increasing trend in the number of incidence cases (Figure 14A). And the TBL mortality projections for women of childbearing age from 2021 to 2035 are similar to the incidence rates, again with an overall downward trend in TBLASMR for women of childbearing age from 2021 to 2035, but with an overall increasing trend in the number of morbidities (Figure 14B). These results indicate a decreasing trend in both ASIR and ASMR trends for TBL in women of reproductive age, but an increasing trend in the number of morbidities and deaths.

**Figure 14:**
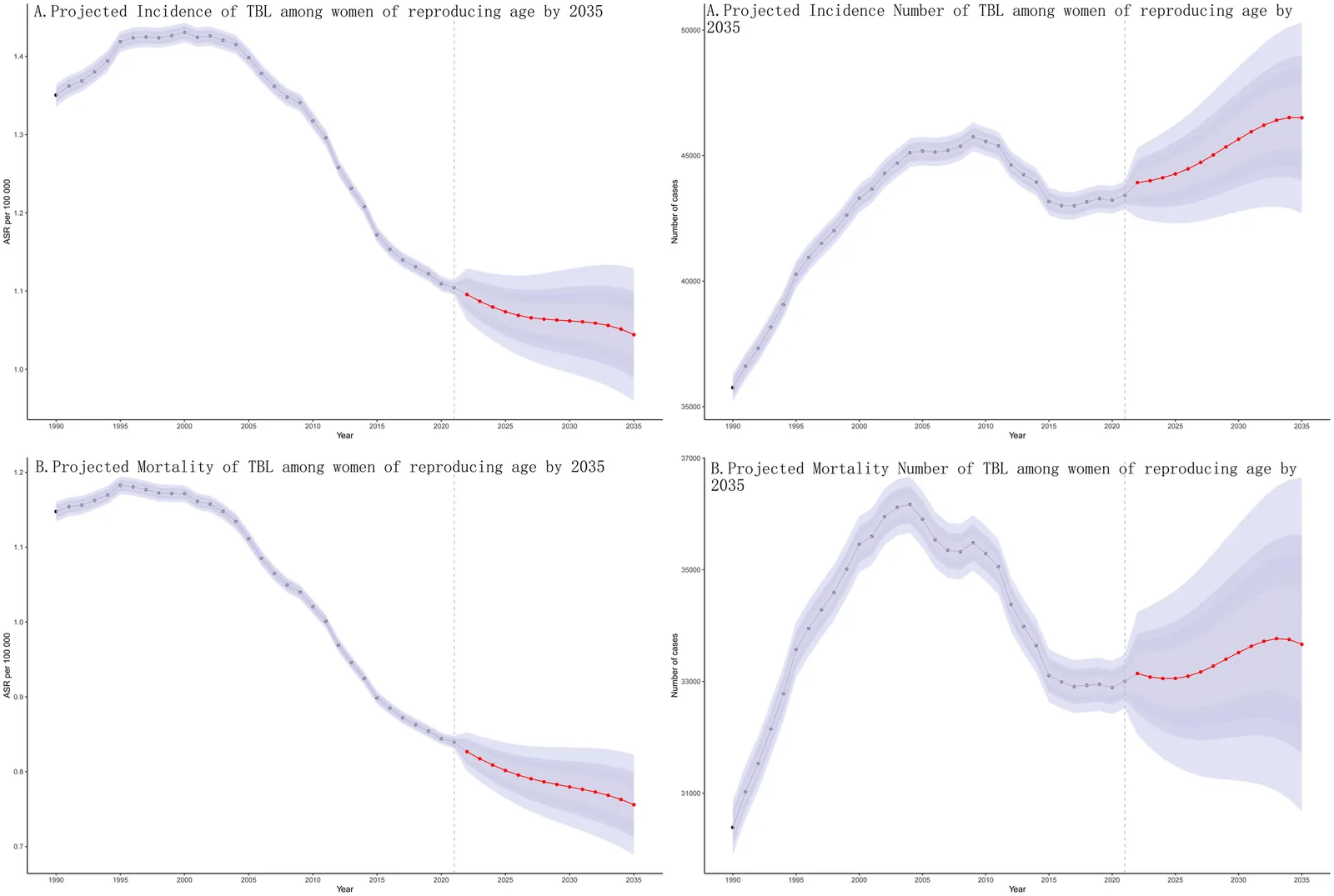
Projected incidence and mortality of TBL among women of reproductive age by BAPC through 2035.

## Discussions

This study found that the incidence, mortality, and DALY rates of TBL among women of reproductive age worldwide all followed a “rise-then-fall” trajectory—with incidence peaking in 2001, mortality peaking in 2000, and DALYs in 1996. All three indicators showed the most significant decline between 2010 and 2016, and by 2021, they had all fallen significantly below 1990 levels. This trend is the result of the combined effects of multiple factors and reflects the complex interactions among tobacco control, environmental governance, advances in medical technology, and demographic changes.

Smoking is the primary modifiable risk factor for lung cancer. The smoking rate among men began to decline immediately after World War II, while the smoking rate among women began to rise after the war and started to decline in the late 1960s; as a result, the peak in lung cancer incidence among women occurred significantly later than among men (7, 29). The finding in this study that the incidence of TBL among women of childbearing age peaked in 2001 is consistent with this historical context. The implementation of tobacco control policies has yielded significant public health benefits—a study covering 30 European countries estimated that, under the most stringent tobacco control policies, 1.65 million cases of lung cancer could be prevented over a 20-year period, with a higher proportion of preventable cases among women (23.2%) than among men (19.8%) (30). However, it is worth noting that the global smoking rate among women is declining more slowly than that among men, and women exhibit greater biological susceptibility to tobacco carcinogens—female smokers face approximately three times the risk of lung cancer compared to men, have higher levels of DNA adducts in their lung tissue, and are more prone to p53 and K-RAS gene mutations (31). The biological basis for this susceptibility is mediated by multiple synergistic mechanisms: First, estrogen can directly synergize with tobacco carcinogens (such as benzo[a]pyrene); cigarette smoke stimulates the metabolism of estradiol into the genotoxic 4-hydroxyestrogen, which forms DNA adducts with the p53 and K-ras genes; Second, the estrogen receptor alpha (ERα) can upregulate the expression of CYP1A1 and CYP1B1, enhancing the metabolic activation of tobacco polycyclic aromatic hydrocarbons; CYP1A1 expression in the lungs of female smokers can be 3.9 times higher than in male smokers; furthermore, women's relatively weaker DNA repair capacity further exacerbates the accumulation of gene mutations. These mechanisms collectively explain why women face a higher risk of lung cancer even at lower levels of smoking exposure (14, 32–34). This explains why the rate of decline in TBL incidence among men is approximately twice that among women, and suggests that women of childbearing age should receive more targeted strategies in smoking cessation interventions.

For women of childbearing age, the incidence of TBL is not driven solely by smoking. Globally, about one-third of households use biomass or coal for cooking, heating, and lighting, and women and children are the groups most severely exposed to indoor air pollution (35). In China, although the smoking rate among women is low (<5%), as many as 81.65% of female lung cancer cases are attributed to risk factors other than smoking; outdoor air pollution and the burning of solid fuels indoors are considered major contributing factors (36, 37). Women of childbearing age—especially those in low- and middle-income countries—spend long hours each day in the kitchen and near the stove because they are primarily responsible for cooking and heating their homes (38). An exposure assessment study in India showed that among solid fuel users, the highest 24-hour average exposure concentrations were observed among women responsible for cooking, with women aged 15–40 having the highest exposure levels because they were most likely to be involved in cooking or assisting with cooking; in contrast, postmenopausal women had relatively lower exposure levels, likely because their cooking activities decreased with age or because they had migrated to urban areas and switched to cleaner energy sources (39). In addition, indoor air pollution has a more significant negative impact on sex hormone levels in premenopausal women, which may further increase their susceptibility to lung cancer (40). Respiratory cancers (including nasopharyngeal cancer and lung cancer) are strongly linked to coal-burning pollution (41). Systematic reviews and meta-analyses have confirmed that the risk of lung cancer associated with solid fuel use is significantly higher among women (OR 1.81) than among men (OR 1.16) (42). In this study, the incidence of TBL among women of childbearing age began to decline after the start of the 21st century, which may partly reflect the effectiveness of policies in various countries regarding the promotion of clean energy and improvements in air quality.

The widespread adoption of early screening and diagnostic technologies for TBL has had a complex dual effect on incidence statistics. On the one hand, low-dose spiral CT screening has significantly increased the detection rate of stage I lung cancer (RR = 2.93) while reducing lung cancer mortality by 16% (RR = 0.84); moreover, the benefits appear to be greater for women (RR = 0.69) than for men (RR = 0.86) (7, 43). The NELSON trial further showed that LDCT screening reduced the 10-year cumulative mortality rate from lung cancer by 33% in women, compared with a 24% reduction in men (44). These technological advances have led to the earlier detection of TBL cases, causing a short-term spike in incidence statistics; at the same time, however, early treatment has resulted in a corresponding decline in mortality rates, and the disease burden has been reduced thanks to more effective interventions. In China, one-time low-dose CT (LDCT) screening has been shown to reduce lung cancer mortality by 31% among high-risk populations, providing a viable approach for implementing large-scale screening in resource-limited areas (45). In this study, the concurrent and accelerating decline in incidence, mortality, and DALY rates from 2010 to 2016 was the result of the combined effects of advances in diagnostic technology, innovations in treatment methods (such as the application of targeted therapy and immunotherapy), and primary prevention policies.

A notable finding in this study is that while the number of cases among women aged 35–49 is on the rise, the incidence rate is declining; furthermore, although the DALY rate in this age group is decreasing, the absolute number of DALYs is increasing, particularly among women aged 40–49, where DALYs increase with age. This apparent contradiction—where the incidence rate declines while the disease burden increases—reflects a structural characteristic in which the rising number of people with the disease coexists with a reduced individual risk of developing it, against the backdrop of population growth and aging. From a biological perspective, as people age, lung tissue function gradually declines, immune function weakens, and the ability to clear carcinogens diminishes. Additionally, because hormone levels in women fluctuate significantly with the reproductive cycle, estrogen plays a crucial role in the development and progression of lung cancer—estrogen can interact synergistically with tobacco compounds, induce the expression of metabolic enzymes, promote the production of reactive oxygen species and the formation of DNA adducts, and thereby drive the development of lung cancer (46–48).

From a sociological perspective, the age range of 35–49 is a critical period during which women assume multiple roles within their families and society. Women in this age group may participate more frequently in early screening due to increased health awareness, but they may also face issues such as unequal distribution of health resources, the cumulative effects of occupational exposure, and an excessive burden of family caregiving. Therefore, the prevention and control of TBL in this age group must address both biological risk screening and social support interventions.

Further analysis stratified by the Socio-Demographic Index (SDI) revealed that in 2021, both the incidence and mortality of TBL among women of childbearing age in high-SDI regions showed a downward trend, whereas incidence and mortality rates continued to rise in low-SDI and lower-middle-SDI regions. As a comprehensive indicator of socioeconomic development (including per capita income, average educational attainment, and total fertility rate), the SDI exhibits a complex, nonlinear relationship with the burden of TBL. This relationship provides important stratified guidance for the formulation of targeted prevention and control strategies on a global scale.

In regions with high SDI, the incidence, mortality, and DALY rates among women of reproductive age began to decline at an accelerating rate around 2010; by 2021, the absolute number of DALYs had also decreased, making this the only SDI group globally to achieve a decline in the absolute number of DALYs. According to the GBD 2021 global data, the ASDR burden is most concentrated in countries and regions with high SDI, and there is a significant positive correlation between SDI and ASDR. However, the EAPC showed the lowest DALY rate in regions with high SDI. Some researchers suggest that this may be because regions with high SDI already have a solid foundation, and greater efforts are needed to achieve more significant results (19). In the field of public health, this phenomenon is known as the law of “diminishing marginal returns”—once the disease burden has been reduced to a relatively low level, further reductions require more targeted and costly interventions (49). Furthermore, the incidence and prevalence of TBL in high-SDI regions continue to rise, driven by population growth and aging. GBD 2021 projections indicate that while the age-standardized incidence of TBL will continue to decline, the number of new cases, prevalence, deaths, and DALYs will still increase by 2035. Therefore, policymakers should not succumb to a false sense of “mission accomplished” simply because a downward inflection point has been reached in high-SDI regions, but should continue to strengthen tobacco control and screening strategies specifically targeting women.

In sharp contrast to regions with high SDI, regions with low and lower-middle SDI all show rising trends in TBL incidence, mortality, and DALY rates among women of childbearing age; moreover, regions with lower-middle SDI have the highest EAPC, reflecting that these regions are currently experiencing the “accelerated phase of the lung cancer burden” during their epidemiological transition. A decomposition analysis further revealed systematic differences in the primary drivers of the increase in the TBL burden across different SDI groups: in low-SDI countries, the increase in the disease burden was primarily driven by population growth; in middle-SDI countries, it was primarily driven by population aging; and in high-SDI countries, it was primarily driven by epidemiological transition (i.e., changes in patterns of exposure to risk factors). This finding is crucial for developing targeted intervention strategies—regions with low SDI must address the rapid growth in total population and the resulting surge in cases, while regions with medium SDI need to pay particular attention to the cumulative effects of the TBL burden among older women. The key drivers behind the continued rise in the burden of TBL in low-SDI and lower-middle-SDI regions include: Multinational tobacco companies shifting their marketing focus to low-income countries, resulting in rising rather than falling smoking rates among women in these regions; approximately one-third of households still relying on biomass or coal for cooking and heating, leading to persistent exposure to indoor air pollution; severe shortages of medical resources, causing most TBL cases to be diagnosed at an advanced stage; and a lack of awareness among women of childbearing age regarding the early symptoms of TBL, leading to widespread delays in seeking medical care (35, 50–52). It is worth noting that the EAPC is highest in regions with low to medium SDI scores, which partly reflects the fact that these regions have begun to strengthen their disease prevention and control capabilities and increase investment in medical resources in recent years. However, given their weak foundational capacity, improvements in diagnostic capabilities may actually lead to the detection of more previously undiagnosed cases in the short term, thereby inflating the reported incidence rates (53, 54). This phenomenon suggests that when interpreting rising incidence trends in regions with low to moderate SDI, it is necessary to carefully distinguish between “a genuine increase in incidence” and “a statistical increase resulting from improved diagnostic capabilities.”

In 2021, among the 21 GBD regions worldwide, both the incidence and mortality rates of TBL among women of childbearing age (45–49 years) showed significant regional heterogeneity. East Asia has the highest incidence and mortality rates in the world, a situation closely linked to the region’s rapid economic development (particularly in China), the Westernization of lifestyles, low smoking rates among women but high exposure to secondhand smoke, and indoor air pollution (such as cooking fumes and the use of solid fuels) (55, 56). It is worth noting that while the incidence rate in sub-Saharan Africa is low, the mortality rate continues to rise, suggesting the existence of a “low incidence, high mortality” paradox in the region—that is, delayed diagnosis, poor access to treatment, and a lack of medical resources have resulted in extremely low survival rates (57). From a temporal perspective, between 1990 and 2021, South Asia had the highest EAPC (estimated annual percentage change), reflecting a rapidly rising burden of TBL in the region, which may be associated with increased tobacco consumption, worsening air pollution, and the lack of screening systems (58, 59); In contrast, the EAPC was lowest in high-income North America, owing to early screening (such as low-dose spiral CT), tobacco control policies (such as high tobacco taxes and smoking bans in public places), and the widespread adoption of targeted treatments (60, 61).

This study further confirms that the SDI (Socio-Demographic Index) exhibits an inverted U-shaped relationship with the incidence and mortality rates of TBL, rising initially and then declining. When the SDI is <0.8, the incidence rate rises as the SDI increases. This is attributed to increased environmental pollution and occupational exposure (e.g., to asbestos, arsenic, and chromium) resulting from industrialization during the early stages of economic development, coupled with improved medical screening capabilities leading to the detection of more cases (62, 63); When the SDI is >0.8, the incidence begins to decline, reflecting the combined effects of the widespread adoption of healthy lifestyles, the effectiveness of tobacco control policies, and the widespread availability of early diagnosis and targeted therapies (such as EGFR and ALK inhibitors) (61, 64). However, it is important to note that in some regions with high SDI scores (such as Western Europe and North America), the rate of decline in the incidence of TBL among women is slowing, suggesting that non-smoking-related factors (such as hormones, genetics, and air pollution) may become new blind spots in prevention and control efforts.

The results of this study indicate that the overall disease burden, incidence, and mortality rates exhibit significant heterogeneity across 204 countries. Countries with high Social Development Index (SDI) scores tend to have higher incidence and mortality rates, while low-SDI countries, such as Nigeria, experience poorer health outcomes due to environmental risk factors (unsafe water sources, poor sanitation, malnutrition, and air pollution) (65). Greenland's high incidence rate is associated with specific disease patterns under its extreme environmental conditions and targeted healthcare services (66); Monaco's high mortality rate, meanwhile, indicates a heavy and growing burden of TBL (tracheal, bronchial, and lung cancer) (67). Due to its large population base, China ranks high in both incidence and mortality rates, influenced by multiple factors including environmental pollution, smoking, genetics, and lifestyle (68, 69). These findings are of significant value for the formulation of stratified, precision-oriented public health policies, with particular attention needed for the specific population of women of childbearing age—who are both a vulnerable group at risk of health issues and key influencers of the next generation's health.

The results of this study indicate that between 1990 and 2021, mortality inequality in TBL among women of childbearing age, stratified by the Social Demographic Index (SDI), decreased: The concentration index (CI) decreased from 0.14 (−0.09, 0.53) to 0.11 (−0.07, 0.25), while the gap in cumulative mortality rates between regions with the highest and lowest SDI also narrowed. This positive trend may be attributed to the gradual improvement in the global distribution of healthcare resources, the expansion of basic health service coverage, and the widespread implementation of health promotion programs. However, the confidence intervals for the CI remain wide, and the disparity in the burden of mortality between regions with low SDI and those with high SDI persists, suggesting that the issue of inequality is far from resolved (70, 71). In regions with low SDI, women of reproductive age face a higher risk of preventable mortality due to a lack of screening facilities, delayed diagnosis, and limited access to treatment; whereas in regions with high SDI, despite abundant resources, lifestyle-related risk factors—such as obesity, sedentary behavior, and late childbearing—continue to contribute to a high burden of TBL (72, 73). This “two-track dilemma” requires policymakers to avoid a “one-size-fits-all” approach and instead design actionable, targeted, and tiered intervention strategies based on SDI stratification and projected trends. In summary, the narrowing of inequalities from 1990 to 2021 demonstrates the potential of global action, but the current uncertainty and residual gaps warn us that without precise strategies tailored to different SDI levels and life stages, inequality may fall into a “low-level equilibrium” trap. Policymakers should seize the window of opportunity presented by these stable and positive trends, shifting resources from “blanket coverage” to “targeted interventions,” thereby achieving an equitable reduction in the TBL mortality burden among women of reproductive age.

This study employed an age-period-cohort model to conduct a systematic analysis of the prevalence trends of TBL among women of childbearing age, revealing distinct epidemiological characteristics of the disease across the three dimensions of age, period, and birth cohort. Analysis of age effects revealed that the prevalence of TBL among women of childbearing age increases steadily with age. After adjusting for period and cohort effects, the 45–49 age group exhibited a higher risk of developing the disease. This finding is consistent with the general pattern of increasing cancer risk with age (74). An analysis of temporal trends reveals a noteworthy pattern: the incidence of TBL has generally declined over the years, suggesting that public health measures implemented in recent years—such as tobacco control policies, improvements in environmental management, and advances in medical technology—may have begun to yield initial results (7). An analysis of cohort effects revealed that the prevalence of the disease was higher in earlier birth cohorts than in later ones, and that the risk of developing TBL decreased with each passing year of birth. This suggests that recent improvements in health education and healthcare have had a positive intergenerational impact on reducing the burden of disease (75). The temporal heterogeneity of these three effects provides a solid evidence-based foundation for developing multi-tiered, targeted public health intervention strategies. The age effect suggests that efforts should focus on establishing targeted screening pathways for the high-risk age group of 45–49-year-olds; the temporal effect indicates that tobacco control policies and environmental management measures have begun to show results, and primary prevention interventions for women of childbearing age should be further strengthened on this foundation; the cohort effect reveals differences in health needs among different birth cohorts, requiring policymakers to develop tiered health management plans based on generational characteristics. The true value of the APC model lies not in providing static epidemiological descriptions, but in empowering public health decision-makers—transforming trend forecasts into precise actions and breaking down macro-level effects into controllable targets—thereby maximizing the reduction of the TBL disease burden under conditions of limited resources.

The decomposition analysis in this study further reveals that the rise in TBL incidence, mortality, and DALY rates among women of reproductive age globally over the past 30 years was not driven by a single factor, but rather resulted from the interplay of population growth, aging, and epidemiological shifts across different regions. Population growth was the primary driver of increased incidence and mortality in the vast majority of low- and middle-income regions (particularly in regions with a middle SDI, as well as in East Asia, Southeast Asia, and South Asia). For example, in East Asia, population growth contributes far more to disease incidence than aging and epidemiological shifts; this is closely linked to the rapid expansion in the absolute number of women of childbearing age in the region, as well as rapid economic development, dramatic changes in lifestyle, and the lag in the redistribution of healthcare resources (76). It is worth noting that population aging plays a particularly prominent role in regions with high SDI (such as high-income North America and Western Europe) and in some regions with medium-to-high SDI (such as Central Europe), as evidenced by the significant positive extension of the aging segment in the decomposition bar charts of deaths and DALYs. This implies that in these regions, although the total number of women of reproductive age may be stabilizing, the rising proportion of older women of reproductive age (e.g., those aged 35–49) leads to more complex conditions, higher treatment failure rates, and greater losses in healthy life years (DALYs), presenting new challenges for prevention and control (77, 78). At the same time, epidemiological changes—which primarily reflect the combined effects of interventions such as disease prevention, diagnosis, treatment, and environmental policies—contributed negatively in most regions (as indicated by the bars extending downward in the figure), confirming the global progress made in tobacco control, environmental governance, early screening, and standardized treatment (79, 80). However, it is a cause for concern that in middle-income countries and some nations in East and South Asia, the negative contribution from epidemiological changes has been relatively small, far from sufficient to offset the enormous positive pressure resulting from population growth, leading to a significant increase in the net burden (79). It is worth noting that in certain regions with low SDI (such as Oceania and Central Sub-Saharan Africa), the negative impact of epidemiological changes is nearly equal to or even weaker than the positive impact of population aging, suggesting that the basic health systems in these regions may not yet be capable of effectively addressing the challenges posed by demographic transition (81).

Based on data from 1990 to 2021, a frontier analysis of ASIR, ASMR, ASDR, and SDI reveals that the disease burden of TBL among women of childbearing age shows a marked divergence across countries at different levels of development and is closely associated with changes in the SDI. First, in countries and regions with high SDI scores (such as Monaco, Iceland, the Netherlands, and France), DALY rates and mortality rates showed an overall downward trend between 1990 and 2020. These countries have invested heavily in medical technology, early screening, tobacco control, vaccination, and health education, establishing relatively well-developed public health systems that have effectively reduced the disease burden associated with TBL (82). In contrast, countries with low SDI (such as Somalia, Niger, Malawi, and The Gambia) saw rising rates of DALYs and mortality during the same period. These regions generally face challenges such as a shortage of medical resources, weak public health infrastructure, and insufficient health literacy, resulting in severely lagging capabilities in the prevention, diagnosis, and treatment of TBL (70). Second, the relationship between incidence rates and the SDI is more complex. Incidence rates in some countries with high SDI scores (such as Iceland, the Netherlands, and Ireland) are on a downward trend, indicating that their prevention strategies—such as effective tobacco tax policies and the promotion of healthy lifestyles—have been successful (1, 82). However, in a small number of regions with high SDI, the incidence has not decreased significantly or has fluctuated slightly; this may be related to the cumulative risk of chronic diseases associated with an aging population, environmental exposures (such as air pollution), and detection bias resulting from increased screening rates. Incidence rates have generally risen in countries with low SDI, likely due to accelerated industrialization during the early stages of economic development, increased tobacco consumption, and the spread of unhealthy lifestyles (83). Overall, as the SDI increases, mortality rates and DALYs show a gradual downward trend, indicating that socioeconomic development plays a positive role in reducing the burden of disease. However, there are a few exceptions; for example, mortality rates remain high in some regions with high SDI, which is often linked to severe population aging and rising mortality rates from chronic diseases. Therefore, when formulating prevention and control strategies, it is necessary to fully consider the practical challenges faced by countries with different SDI levels, develop tailored strategies, improve resource utilization efficiency, and maximize the benefits of TBL prevention and control among women of childbearing age (83, 84). In regions with high SDI, efforts should focus on strengthening early screening, targeted treatment, and chronic disease management for the elderly; in regions with moderate SDI, there is a need to strengthen tobacco control legislation, promote vaccination, and expand community health education; in regions with low SDI, priority should be given to ensuring access to basic healthcare resources, improving infrastructure, and raising public awareness of TBL to curb the rising trend in the disease burden.

This study used the Bayesian age-period-cohort (BAPC) model to forecast the ASIR and ASMR for TBL. The results show that in 2021, both indicators exhibited an overall downward trend in most regions worldwide, including high-income countries and some middle-income countries. However, in stark contrast to the downward trends in ASIR and ASMR, the BAPC model predicts that global TBL incidence and mortality will generally rise between 2021 and 2035, particularly in countries with large populations and rapidly aging populations (such as China, India, Brazil, and Indonesia) (85). The key driver lies in global demographic shifts. First, the absolute number of women of childbearing age worldwide continues to grow, particularly in South Asia, sub-Saharan Africa, and some Southeast Asian countries. Even if the individual risk of contracting the disease decreases, the large population base ensures that the absolute number of cases remains high (71). Second, population aging is a global trend—in middle-income and some high-income countries, the age profile of women of childbearing age has shifted upward overall, and the proportion of women in the age group at high risk for TBL (aged 45 and older) has increased significantly (78). A decomposition analysis indicates that population aging is the primary driver of the rise in absolute incidence and mortality rates of TBL, with its effect sufficient to offset or even exceed the decline in age-standardized rates (86). In terms of the age effect, the risk of TBL increases exponentially with age, accelerating significantly after age 45 and peaking after age 85 (87). Consequently, in countries experiencing rapid demographic transitions, the amplifying effect of an aging population of women of childbearing age on the absolute burden is particularly pronounced. This trend will have far-reaching implications for national healthcare and social security systems: the growing number of women of childbearing age with TBL exacerbates the imbalance between supply and demand for diagnostic equipment, hospital beds, and specialized healthcare personnel, particularly in low- and middle-income countries where medical resources are already scarce; Given the long treatment duration and high costs associated with TBL, the rise in the absolute number of patients places enormous pressure on health insurance funds and leads to catastrophic health expenditures for families; an increasing number of families face multiple challenges—including caregiving, financial, and psychological burdens—and require support from more comprehensive social security systems.

### Targeted interventions

The global burden of TBL is unevenly distributed; incidence and mortality rates are declining in high-income countries but rising in low-income countries. Women are underrepresented in clinical trials, and measures are needed to promote equity, diversity, and inclusion (88).

To implement targeted interventions for total breast cancer (TBC) in women of childbearing age, policymakers should establish a tiered, differentiated action framework based on the Social Development Index (SDI). In high-SDI regions, although the disease burden has decreased, a notable gender disparity persists—with incidence rates among women declining more slowly than among men—and the contribution of non-smoking-related risks, such as secondhand smoke exposure, air pollution, and reproductive factors, is on the rise. Therefore, the focus must shift from scale-driven approaches to precision-driven quality improvement. Specific measures include: lowering the screening age to 35–40 years; incorporating gender-specific risk assessment tools (such as family history of lung cancer, secondhand smoke exposure, indoor pollution, age at first birth, and history of hormone therapy) into guidelines to avoid relying solely on pack-years of smoking; establishing a specialized registry for TBL in women of childbearing age to collect data on reproductive history, hormone use, and molecular subtyping; strengthening the gender dimension of tobacco control policies, promoting “smoke-free housing” certification, and utilizing digital tools to deliver personalized health plans.

In SDI regions, the burden of TBL is currently in a critical window of rapid increase, driven primarily by population aging, making this the most cost-effective time for policy intervention. A “Clean Household Energy First” initiative should be implemented, incorporating clean cooking energy into livelihood subsidies, mandating the installation of exhaust systems, and reducing exposure to indoor pollution. Concurrently, an early lung cancer screening network centered on primary care should be established. County-level hospitals should be equipped with low-dose CT scanners and AI-assisted image interpretation, and primary care physicians should be trained to use localized risk scoring cards (covering age, smoking, secondhand smoke exposure, duration of cooking with wood/fuel, and family history). Lung cancer screening should be integrated with the “Two Cancers” (breast and cervical cancer) screening program. Regarding tobacco control, tobacco taxes should be increased, misleading advertisements targeting women should be banned, and “Say No to the First Cigarette” peer education programs should be implemented in schools. In transition-phase regions such as South Asia, a tiered increase in tobacco taxes should be initiated, with the revenue earmarked specifically for screening.

In regions with low SDI, resource constraints are most severe, and the increasing burden is driven by population growth, necessitating the adoption of low-cost, high-impact strategies. Promote community-empowered “Clean Kitchen” demonstration projects, where female leaders teach ventilation methods, and integrate information on the hazards of smoke into rural radio broadcasts and maternal and child health home visits. Establish symptom-based early diagnosis and referral pathways for women of childbearing age, train village doctors to recognize warning signs (persistent cough, bloody sputum, chest pain), and open fast-track referral channels. Actively seek international aid to procure low-dose CT scanners and GeneXpert devices, and establish sister hospital partnerships with high-SDI countries to conduct telemedicine consultations. For “low-incidence, high-mortality” regions such as Western Sub-Saharan Africa, establish regional diagnostic and referral networks; procure targeted therapies through centralized purchasing by the African Union Medicines Regulatory Authority and establish a special fund to reduce out-of-pocket costs.

In addition to SDI stratification, interventions should be optimized by considering age, period, and cohort effects. The 45–49 age group has a significantly higher risk and should be prioritized for screening resources, with dedicated pathways and weekend clinics established. The period effect indicates a declining incidence trend; therefore, tobacco control policies should be strengthened, smoking cessation counseling and nicotine replacement therapy should be included in health insurance coverage, and an environmental risk early warning system should be established. The cohort effect suggests that retrospective screening and regular follow-up should be conducted for earlier birth cohorts (aged 50 and above), while for later birth cohorts (under 30), TBL prevention should be incorporated into secondary school health curricula, and digital tools should be utilized to interrupt the intergenerational transmission of unhealthy lifestyles.

Cross-regional strategies include: establishing a dynamic TBL monitoring platform for women of childbearing age; integrating multi-source data to predict incidence trends; updating the inequality dashboard based on SDI deciles; and triggering international assistance when the mortality ratio exceeds 3 times. Prioritize fertility preservation by noting the impact on ovarian function in all treatment guidelines and providing bundled subsidies for services such as egg/embryo freezing; establish peer support groups and psychological counseling hotlines to reduce stigma. Regarding funding, incorporate TBL screening into basic public health programs, implement high-reimbursement rates for high-risk groups aged 45–49, and establish a drug buffer stock. Set annual inequality reduction targets (e.g., a 0.005 annual decrease in the concentration index); pilot tiered randomized controlled trials in regions with medium SDI scores; upon validation, scale up to regions with low SDI scores; and establish a negative list mechanism. By precisely identifying the dominant factors in different regions—implementing population-based prevention in areas with rapid population growth, improving service quality in aging regions, and addressing weaknesses and strengthening foundations in underserved areas—we can overcome demographic pressures and achieve a substantial reduction in the global TBL burden among women of reproductive age.

## Limitations

This study has certain limitations that must be fully considered when interpreting the results. First, the study relies strictly on aggregated, population-level data provided by the Global Burden of Disease (GBD) study; therefore, it inevitably faces the risk of the ecological fallacy: associations between the TBL cancer burden and risk factors observed at the population level cannot be directly inferred as causal relationships at the individual level. We strongly caution readers against making individual-level attribution inferences based on the population estimates in this study. Second, GBD data have inherent limitations, particularly regarding significant differences in diagnostic accuracy and data registration quality across regions with varying Socio-Demographic Index (SDI) levels. Regions with high SDI typically have well-established systems for pathological confirmation and cancer registration, whereas regions with low SDI may rely on clinical diagnoses, which can lead to missed or misdiagnosed cases, as well as insufficient registration coverage and non-standardized cause-of-death coding. This affects the reliability of comparisons of global and regional trends. Third, the global cancer registry data relied upon in this study vary in regional coverage and quality, with data from some low-income countries being incomplete; definitions of the age range for women of reproductive age (e.g., 15–49 years or 20–49 years) also differ across databases, limiting the precision of cross-study comparisons. Fourth, although this study discusses risk factors such as smoking, environmental exposure, and reproductive factors, the aggregated nature of the data makes it difficult to quantitatively assess the independent contribution of each factor to the observed trends. Our future research may focus on the following directions: conducting multi-country, large-sample attributable risk studies on TBL cancers among women of reproductive age (e.g., prospective cohorts) to clarify the population-attributable fractions of each factor; utilizing age-period-cohort analysis to decompose the contributions of age, period, and cohort effects to the trends; and conducting intervention studies to evaluate the actual impact of promoting clean energy, integrating LDCT screening, and implementing gender-sensitive tobacco control strategies on reducing the burden of TBL.

## Conclusions

Women of reproductive age worldwide continue to bear a heavy burden of TBL; although the overall burden of TBL among women of reproductive age has decreased globally, it must be acknowledged that certain countries still face challenges in preventing and treating TBL in this population.

## Data Availability

The original contributions presented in the study are included in the article/Supplementary Material, further inquiries can be directed to the corresponding authors.
